# Genetic overlap between functional impairment and depression and anxiety symptom severity: evidence from the GLAD Study

**DOI:** 10.1017/S0033291725101037

**Published:** 2025-08-05

**Authors:** Megan Skelton, Jessica Mundy, Abigail R. ter Kuile, Brett N. Adey, Chérie Armour, Joshua E. J. Buckman, Jonathan R. I. Coleman, Molly R. Davies, Colette R. Hirsch, Matthew Hotopf, Ian R. Jones, Gursharan Kalsi, Georgina Krebs, Sang Hyuck Lee, Yuhao Lin, Andrew M. McIntosh, Alicia J. Peel, Christopher Rayner, Katharine A. Rimes, Daniel J. Smith, Katherine N. Thompson, David Veale, James T. R. Walters, Christopher Hübel, Gerome Breen, Thalia C. Eley

**Affiliations:** 1 Institute of Psychiatry, Psychology, and Neuroscience, King’s College London, London, UK; 2 National Institute for Health and Care Research (NIHR) Maudsley Biomedical Research Centre, South London and Maudsley NHS Foundation Trust, London, UK; 3 Research Centre for Stress, Trauma & Related Conditions (STARC), School of Psychology, Queen’s University Belfast, Belfast, Northern Ireland, UK; 4 Centre for Outcomes Research and Effectiveness (CORE), Research Department of Clinical, Educational and Health Psychology, University College London, London, UK; 5 iCope – Camden and Islington Psychological Therapies Services, Camden and Islington NHS Foundation Trust, London, UK; 6 Chief Academic Officer for South London and Maudsley NHS Foundation Trust, London, UK; 7 National Centre for Mental Health, Division of Psychiatry and Clinical Neuroscience, Cardiff University, Cardiff, UK; 8 Division of Psychiatry, Centre for Clinical Brain Sciences, University of Edinburgh, Edinburgh, UK; 9 Department of Medical Epidemiology and Biostatistics, Karolinska Institutet, Stockholm, Sweden; 10 National Centre for Register-based Research, Aarhus Business and Social Sciences, Aarhus University, Aarhus, Denmark

**Keywords:** dimensional symptoms, disability, genetic correlations, GWAS, internalizing symptoms, mental health

## Abstract

**Background:**

Functional impairment in daily activities, such as work and socializing, is part of the diagnostic criteria for major depressive disorder and most anxiety disorders. Despite evidence that symptom severity and functional impairment are partially distinct, functional impairment is often overlooked. To assess whether functional impairment captures diagnostically relevant genetic liability beyond that of symptoms, we aimed to estimate the heritability of, and genetic correlations between, key measures of current depression symptoms, anxiety symptoms, and functional impairment.

**Methods:**

In 17,130 individuals with lifetime depression or anxiety from the Genetic Links to Anxiety and Depression (GLAD) Study, we analyzed total scores from the Patient Health Questionnaire-9 (depression symptoms), Generalized Anxiety Disorder-7 (anxiety symptoms), and Work and Social Adjustment Scale (functional impairment). Genome-wide association analyses were performed with REGENIE. Heritability was estimated using GCTA-GREML and genetic correlations with bivariate-GREML.

**Results:**

The phenotypic correlations were moderate across the three measures (Pearson’s *r* = 0.50–0.69). All three scales were found to be under low but significant genetic influence (single-nucleotide polymorphism-based heritability [*h*^2^_SNP_] = 0.11–0.19) with high genetic correlations between them (*r_g_* = 0.79–0.87).

**Conclusions:**

Among individuals with lifetime depression or anxiety from the GLAD Study, the genetic variants that underlie symptom severity largely overlap with those influencing functional impairment. This suggests that self-reported functional impairment, while clinically relevant for diagnosis and treatment outcomes, does not reflect substantial additional genetic liability beyond that captured by symptom-based measures of depression or anxiety.

## Introduction

Major depressive disorder (MDD) and anxiety disorders are characterized by emotional symptoms, including low mood, excessive worry, and fear, which can cause clinically significant distress or impairment in important areas of functioning. Functional impairment refers to difficulties performing tasks and roles, such as work or social activities, and is a critical factor in distinguishing normal symptom variation from diagnostic conditions. Patients rate a return to normal functioning as an important treatment outcome (Zimmerman et al., 2006). Despite this, in both clinical and research contexts, definitions of remission and recovery often rely on symptom severity scales that typically do not explicitly measure impairment (Kamenov, Cabello, Coenen, & Ayuso-Mateos, 2015). While individuals experiencing no symptoms will, by extension, not experience functional impairment due to symptoms, beyond this, individuals with the same level of symptom severity can experience different levels of functional impairment (Denninger et al., 2011; Zimmerman et al., 2008). Phenotypic correlations between total symptom scores and measures of functional impairment are, therefore, typically moderate (e.g., *r* = 0.43–0.63; Kroenke, Spitzer, & Williams, 2001; Spitzer, Kroenke, Williams, & Löwe, 2006; Zahra et al., 2014). Furthermore, some patients considered to be in remission report persistent impairment from residual symptoms (IsHak et al., 2016; Saris et al., 2017). These findings highlight the importance of assessing impairment alongside symptoms for a more complete and accurate reflection of patient well-being and treatment efficacy.

MDD and anxiety disorders show moderate heritability, defined as the proportion of phenotypic variance due to genetic variation in the population, with twin-based estimates of approximately 25–40% (Hettema, Neale, & Kendler, 2001; Sullivan, Neale, & Kendler, 2000). Heritability estimates from genetic variant-level analyses, known as genome-wide association studies (GWASs), are lower, roughly 5–20% (Cross-Disorder Group of the PGC, 2013; Purves et al., 2020), as they capture only the additive effects of common genotyped variants rather than all genetic influences. This heritability is explained by many genetic variants, each with a very small effect size (Purves et al., 2020; Wray et al., 2018). The substantial genetic overlap between MDD and anxiety disorders is well-established; genetic correlation (*r_g_*) estimates typically range from 0.8 to 1 (Kendler et al., 1992; Purves et al., 2020).

Research into the genetic influences on functional impairment is much more limited (McGrath et al., 2013; Ordonana et al., 2013). Twin studies suggest a moderate heritable component (20–30%) (Rijsdijk et al., 2003; Romeis et al., 2005). One twin study found that, while most genetic influences on functional impairment were shared with MDD, a modest proportion (14%) were specific to impairment (Foley et al., 2003). However, genomic data have not successfully been used to estimate the heritability of impairment, and genetic correlations between symptoms and impairment remain unclear. Moderate genetic correlations between symptoms and impairment, mirroring phenotypic correlations (Waszczuk, Zavos, Gregory, & Eley, 2014), would indicate a shared genetic liability alongside symptom-specific and impairment-specific genetic influences.

To maximize sample sizes, some GWAS have used current symptom scores as depression or anxiety phenotypes (Direk et al., 2017; Levey et al., 2020). While there is evidence of high genetic correlations between symptoms and disorder phenotypes (Direk et al., 2017; Levey et al., 2020; Purves et al., 2020), more recent analyses in the UK Biobank reported lower correlations between current and lifetime worst-episode depression symptoms (between 0.43 and 0.87; Huang et al., 2023). Impairment-specific genetic influences could capture a liability, beyond that for current symptoms, that is relevant to full diagnostic presentations of depression and anxiety. Genetic correlation estimates could clarify whether there is value in supplementing symptom scales with measures of functional impairment in genetic studies of depression and anxiety. Furthermore, as genetic information is increasingly explored as a prognostic predictor, the extent of the correlation could indicate whether supplementing genetic information on symptom severity with that on impairment may improve predictive accuracy.

We investigated the genetic influences on self-reported measures of current depression symptoms (Patient Health Questionnaire 9-item version [PHQ-9]) (Kroenke et al., 2001), anxiety symptoms (Generalized Anxiety Disorder 7-item scale [GAD-7]) (Spitzer et al., 2006), and functional impairment (Work and Social Adjustment Scale [WSAS]) (Marks, 1986). In a sample of individuals with lifetime depression or anxiety, we estimated single-nucleotide polymorphism (SNP)-based heritability (*h*^2^_SNP_) and genetic correlations between these measures. To better understand the genetic characteristics of functional impairment, we also estimated genetic correlations with selected external phenotypes. Understanding the genetic influences on these measures and the relationships between them is important for interpreting findings in studies where they are used. The PHQ-9 and GAD-7 are endorsed by research funders and academic journals as standard measures of adult depression and anxiety (Farber, Gage, Kemmer, & White, 2023; Wellcome, 2020). Furthermore, the PHQ-9, GAD-7, and WSAS are core outcome measures in the National Health Service (NHS) England ‘Talking Therapies for anxiety and depression’ program (formerly ‘IAPT’), with the symptom scales used to define recovery and improvement (The National Collaborating Centre for Mental Health, 2023). We expected moderate genetic correlations significantly different from zero (0.4–0.7) between symptoms and impairment, reflecting existing phenotypic estimates (Kroenke et al., 2001; Spitzer et al., 2006; Zahra et al., 2014).

## Materials and methods

### Sample

This analysis used a sample of participants from the Genetic Links to Anxiety and Depression (GLAD) Study. GLAD is an online study recruiting individuals primarily from the general UK population, aged 16 years and older, with lifetime experience of depression and/or anxiety (Davies et al., 2019). Participants were, therefore, more likely to have nonzero symptom scores, allowing us to investigate associations with impairment across a full distribution of severities. GLAD participants provide informed consent before completing an online sign-up questionnaire, which includes assessments of clinical and demographic information. Participants are required to meet the case criteria on diagnostic questionnaires or self-report a diagnosis by a medical professional. They are then sent a saliva sample collection kit with which they provide their genetic data. Almost all (96%) participants have received treatment for their depression or anxiety, the majority have recurrent depression, and over half have experienced an anxiety disorder (Davies et al., 2019). The analysis was centered around three phenotypes as described below: depression symptoms, anxiety symptoms, and functional impairment. Our analysis was limited to participants with phenotypic data collected during the sign-up questionnaire for at least one of these measures, covariate information, and genotype data that passed quality control (*N* = 17,130; range across phenotypes = 17,081–17,107). Ethical approval for the GLAD Study was obtained from the London–Fulham Research Ethics Committee (REC reference: 18/LO/1218). The authors assert that all procedures contributing to this work comply with the ethical standards of the relevant national and institutional committees on human experimentation and with the Declaration of Helsinki 1975, as revised in 2013.

### Phenotype measures

*Depression symptoms* were assessed using the PHQ-9 (Supplementary Information 1), which measures the recent frequency of nine symptoms using the stem question: ‘Over the last 2 weeks, how often have you been bothered by any of the following problems?’ Each item has a four-point response scale from ‘not at all’ (scored 0) to ‘nearly every day’ (scored 3). Summed scores indicate severity from 0 to 27. The PHQ-9 had good internal reliability in the GLAD sample (*α* = 0.90) and has demonstrated good test–retest reliability in other studies (intraclass correlation = 0.84) (Kroenke et al., 2001).

*Anxiety symptoms* were assessed by the GAD-7 (Supplementary Information 1), which has a similar format to the PHQ-9. It has the same overarching question regarding the frequency of recent problems, with seven anxiety symptoms rated on the four-point scale, yielding total scores from 0 to 21. Internal consistency in the GLAD sample was good (*α* = 0.91), and good test–retest reliability has been reported (intraclass correlation = 0.83) (Spitzer et al., 2006).

The development papers for the PHQ-9 and GAD-7 (Kroenke et al., 2001; Spitzer et al., 2006) presented the symptom scales alongside a functional impairment item to validate their use (see Supplementary Information 1). This item was not included for either measure in the GLAD Study, and is not consistently used across clinical settings (e.g., NHS Talking Therapies for anxiety and depression) or research settings. Even when the impairment item is present, it is not incorporated into the total PHQ-9 and GAD-7 symptom scores used to define clinical outcomes.

The WSAS assesses the impact of symptoms on daily living (**functional impairment**) across the following five domains: the ability to work, home management, social leisure activities, private leisure activities, and the ability to form and maintain close relationships. Each item is worded as, “because of my problem my <domain> is impaired.” A nine-point response scale of not at all (scored 0) to very severely (scored 8) gives total scores from 0 to 40. The WSAS showed good internal consistency in GLAD (*α* = 0.85). In another sample, the WSAS was captured by a single factor and demonstrated acceptable test–retest reliability (0.73) (Mundt, Marks, Shear, & Greist, 2002). One limitation is that the ‘ability to work’ item could be answered ‘not applicable’ if respondents were not in employment or education. The subsequent missing data can be handled by imputation using the mean of the individual’s four nonmissing WSAS items (as done in NHS Digital), but this can introduce bias and lead to spurious results if the data are missing ‘not at random’ (Little & Rubin, 2002). We explored a complete case of the total WSAS sum score from all five items and a four-item WSAS sum score excluding the work item. This exploration, presented in Supplementary Information 2, included phenotypic analyses (Cronbach’s alpha and group comparisons) and genetic correlations between each WSAS sum score and the work item. Subsequently, we present the results from an individual mean imputed WSAS sum score (as per NHS Digital), with results from sensitivity analyses using the complete case and four-item sum scores in the Supplementary Materials.

### Genotyping and quality control

Genotyping was performed by ThermoFisher on behalf of the National Institute for Health and Care Research (NIHR) Cambridge Biomedical Research Centre using the Affymetrix UK Biobank Axiom Array. The dataset used was from Freeze 2.0. Genetic quality control, further detailed in Supplementary Information 3, was conducted in PLINK v1.9. (Chang et al., 2015) by applying the following exclusion thresholds for individuals: >5% missing variants, non-European genetic ancestry (as specific ancestry groups were insufficiently sized for analysis), and signs of potential genotyping error or contamination (global identity by descent outliers, discordant reported sex at birth, and genetically inferred sex). The sample comprised 18,349 individuals before quality control and 17,147 afterward (1,202 were removed), with further exclusions for missing phenotype data resulting in an analysis sample of 17,130. Genetic variants were excluded if they had missingness >2%, minor allele frequency < 1%, or Hardy–Weinberg equilibrium *p* < 1 × 10^−8^. Genotype imputation was performed using the TOPMed reference panel (version r2 on GRCh38; Das et al., 2016; Fuchsberger, Abecasis, & Hinds, 2014; Taliun et al., 2021). Quality control filters were applied both before and after imputation, with an additional post-imputation quality threshold of *R*^2^ > 0.3. A total of 7,027,957 variants remained for analysis.

### Statistical analyses

A GWAS was performed with each phenotype using REGENIE (version 2.2.4; Mbatchou et al., 2021) under a linear model. We included covariates that could act as confounders or explain variance in the phenotypes (Salk, Hyde, & Abramson, 2017; Sutin et al., 2013). In linear regression, this can yield more precise SNP effect estimates and increase power (Mefford & Witte, 2012). Covariates were age, age^2^, sex (binary), genotyping batch (categorical, four levels), and the first 10 genetic principal components. The *h*^2^_SNP_ was estimated with genomic-relatedness-based restricted maximum-likelihood in ‘genome-wide complex trait analysis’ software (GCTA-GREML, version 1.94; Yang, Lee, Goddard, & Visscher, 2011). GREML methods create a genomic relatedness matrix using individual-level data on common SNPs genotyped on the genetic array. To prevent inflation of the matrix and biased estimates, we followed standard recommendations (Lee et al., 2012; Yang et al., 2011) and excluded one of each pair of participants with genomic relatedness >0.05 (*n* = 373). For all GREML analyses, we used genotyped data and included the same covariates as for the GWAS, described above.

The genetic correlations between the three phenotypes were calculated using GCTA bivariate-GREML (Lee et al., 2012). We tested whether the genetic correlations between symptoms and functional impairment differed from 1 using the ‘*reml-bivar-lrt-rg’* flag in GCTA to perform a likelihood ratio test and generate a *p*-value. This test was also used to produce *p*-values for the default test of difference from *r_g_* = 0. Furthermore, we estimated the proportion of the phenotypic correlation attributable to genetic correlation by performing calculations and simulating standard errors as described previously (de Vries et al., 2021; Morris, Davies, Hemani, & Smith, 2020).

We estimated genetic correlations with 10 prespecified external phenotypes using linkage-disequilibrium score regression (LDSC, version 1.0.1; Bulik-Sullivan et al., 2015; Bulik-Sullivan, Loh, et al., 2015). First, we selected five case–control psychiatric phenotypes: MDD (Wray et al., 2018), anxiety disorders (Purves et al., 2020), schizophrenia (Trubetskoy et al., 2022), attention-deficit and hyperactivity disorder (ADHD; Demontis et al., 2023), and post-traumatic stress disorder (PTSD; Stein et al., 2021). See Supplementary Table 1 for further details of the source studies. Genetic correlations with MDD and anxiety disorders would reveal shared genetic influences between our symptom and impairment measures and phenotypes that incorporate diagnostic elements beyond symptom severity, including impairment. The remaining case–control phenotypes were selected to investigate whether the genetic influences on anxiety- or depression-related impairment were shared with diagnostically distinct disorders. Second, we examined five additional traits, four quantitative and one binary: neuroticism (Gupta et al., 2024), self-rated fatigue (Deary et al., 2018), years of education (Lee et al., 2018), self-rated health (Harris et al., 2017), and smoking (Liu et al., 2019). These were selected for their relevance to our phenotypes. Neuroticism is a risk factor for both anxiety and depression (Fryers & Brugha, 2013), and an analysis of depression symptoms showed that fatigue explained substantial variance in impairment (Fried & Nesse, 2014). Education reflects cognitive and socioeconomic factors, while self-rated health and smoking are each associated with mental and physical health, with smoking representing a health behavior. To formally test whether the genetic correlations with functional impairment differed from those estimated with the symptom measures, we used a block jackknife procedure with 200 blocks. Bonferroni corrections were applied to significance thresholds: *p* < 0.017 for each of the three heritability and internal correlation estimates, *p* < 0.005 for 10 external correlation tests per measure, and *p* < 0.017 for the correlation comparisons. Analysis was conducted within the King’s College London computational research environment (King’s College London, 2023). Data preparation and visualization were performed in R version 4.1.2 (R Core Team, 2021).

## Results

### Sample characteristics

The characteristics of the sample (*N* = 17,130) are presented in Table 1. Participants had moderate current depression symptoms (PHQ-9), mild anxiety symptoms (GAD-7), and moderate functional impairment (WSAS), on average (Kroenke et al., 2001; Mundt et al., 2002; Spitzer et al., 2006; see Supplementary Figure 1 for distributions).

**Table 1.** Characteristics of analysis sample from the Genetic Links to Anxiety and Depression (GLAD) Study (*N* = 17,130)

		Mean (SD); range or *n* (%)
Age		39.5 (14.6); 16–93
Sex	Female	13,365 (78%)
	Male	3,765 (22%)
Ethnicity^a^	White	16,903 (99%)
	Mixed	105 (0.6%)
	Other	93 (0.5%)
	Unknown	29
Employment status	In paid employment or self-employed	10,309 (60%)
	Full or part-time student	2,061 (12%)
	Unable to work due to sickness or disability	1,862 (11%)
	Retired	1,231 (7%)
	Looking after home and/or family, doing unpaid/voluntary work	871 (5%)
	Unemployed	612 (4%)
	None of the above	143 (1%)
	Unknown	41
University degree	Yes	9,786 (57%)
	No	7,341 (43%)
	Unknown	3
Depression symptoms (PHQ–9)		11.2 (6.9); 0–27
	Unknown	49
Anxiety symptoms (GAD–7)		8.9 (5.9); 0–21
	Unknown	33
Functional impairment (WSAS)^b^		16.8 (9.3); 0–40
	Unknown	23

a Self-reported ethnicity. All participants in this analysis sample met genetic quality control criteria for European ancestry.

b Mean-imputed WSAS score used in the analyses. Unknown values reflect participants with >1 missing WSAS item, ineligible for imputation. Descriptives for the WSAS score before imputation: 17.2 (9.2); 0–40, unknown = 2,064 (majority were ‘not applicable’ responses to the work item).

### Heritability estimates

No variants reached genome-wide significance (*p* < 5 × 10^−8^) in the GWAS of any of the three traits (Supplementary Figure 2). SNP-based heritability estimates were significant (*p* < 0.017) for depression symptoms (0.19, SE = 0.04, *p* = 6 × 10^−9^), anxiety symptoms (0.17, SE = 0.03, *p* = 2 × 10^−7^), and functional impairment (0.11, SE = 0.03, *p* = 2 × 10^−4^).

### Phenotypic and genetic correlations between traits

**Figure 1.**
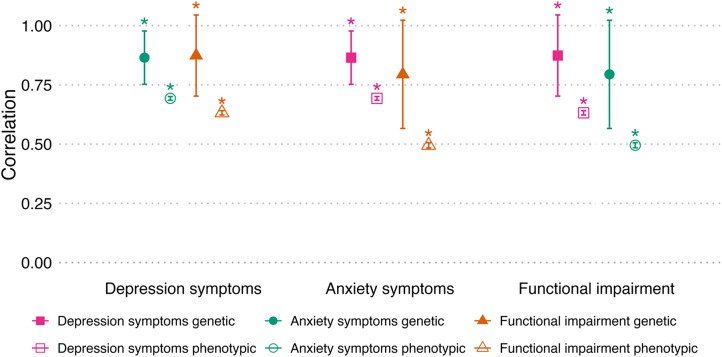
Genetic and phenotypic correlations between depression symptoms, anxiety symptoms, and functional impairment in a sample from the GLAD Study (*N* = 17,130). *Note:* Error bars represent 95% confidence intervals. *Significant at p < 0.017. Depression symptoms = PHQ-9 score, anxiety symptoms = GAD-7 score, functional impairment = WSAS score. Genetic correlations were estimated using GCTA bivariate-GREML and phenotypic correlations using Pearson’s r. For ease of comparability, both sides of the correlations are presented; therefore, information is duplicated. For example, the depression symptoms–functional impairment genetic correlation is presented by the filled orange triangle above ‘Depression symptoms’ on the x-axis and the filled pink square above ‘Functional impairment’.

Phenotypic and genetic correlations between depression symptoms, anxiety symptoms, and functional impairment are presented in Figure 1 (and Supplementary Tables 2 and 3). Phenotypic correlations between traits were significantly different from zero (at *p* < 0.017) and moderate (*r* = 0.50–0.69), with the highest correlation observed between depression and anxiety symptoms and the lowest between anxiety symptoms and functional impairment.

The genetic correlation between depression symptoms and functional impairment was 0.87, which was significantly different from zero (at *p* < 0.017 and *p =* 1.5 × 10^−6^). For anxiety symptoms and functional impairment, the genetic correlation was 0.79 and significant (*p* = 1.3 × 10^−5^). Although all genetic correlations were higher than their corresponding phenotypic correlations, the lowest correlation, both phenotypically and genetically, was observed between anxiety symptoms and impairment. As the genetic correlations between impairment and depression or anxiety symptoms were strong, we formally tested whether they were significantly different from 1. The results indicated that they were not (*p* = 0.098 and *p* = 0.049, respectively, at *p* < 0.025).

The proportion of phenotypic correlation attributable to common genetic variants shared between functional impairment and depression symptoms was 0.20 (95% CI = 0.12–0.27), and for functional impairment and anxiety symptoms was 0.22 (95% CI = 0.12–0.31). This indicated that the measured genetic correlation explained one-fifth of the phenotypic correlation between traits. Using LDSC to estimate genetic correlations produced similar results to those from GCTA-GREML, while heritability estimates were significant but attenuated (Supplementary Information 4), consistent with the reduced power of this summary-statistics-based method (Evans et al., 2018). Phenotypic and genetic explorations of the complete case WSAS with and without the work item showed similar results to the mean-imputed WSAS used in the main analysis and are presented in Supplementary Information 4.

### Genetic correlations with external phenotypes

**Figure 2.**
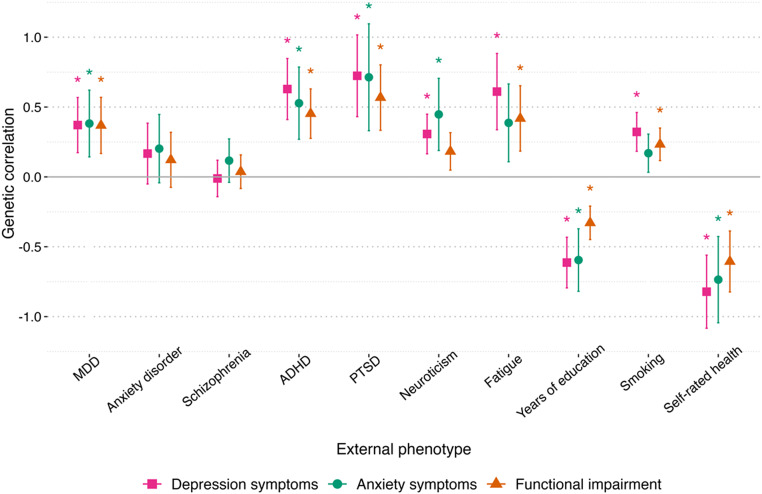
Genetic correlations between depression symptoms, anxiety symptoms, and functional impairment in a GLAD Study sample (*N* = 17,130) and 10 external phenotypes. *Note:* Error bars represent 95% confidence intervals. *Significant at p < 0.005. Depression symptoms = PHQ-9 score, anxiety symptoms = GAD-7 score, functional impairment = WSAS score. MDD = major depressive disorder (Wray et al., 2018), anxiety disorder (Purves et al., 2020), schizophrenia (Trubetskoy et al., 2022), ADHD = attention deficit hyperactivity disorder (Demontis et al., 2023), PTSD = post-traumatic stress disorder (Stein et al., 2021), neuroticism (Gupta et al., 2024), fatigue (Deary et al., 2018), years of education (Lee et al., 2018), smoking (Liu et al., 2019), and self-rated health (Harris et al., 2017). See Supplementary Table 1 for further details of the external phenotypes. Genetic correlations were estimated using LDSC.

LDSC estimates of genetic correlations between each of the measures and 10 external phenotypes are shown in Figure 2 and Supplementary Table 4. All three phenotypes showed nonzero estimates with MDD, ADHD, PTSD, years of education, and self-rated health, which remained significant after correction for multiple testing. Negative correlations with years of education and self-rated health indicated that genetic variants associated with higher symptom or impairment scores were associated with fewer years of education and poorer health ratings. Genetic correlations with neuroticism were significant for depression and anxiety symptoms but not for functional impairment. For both self-rated fatigue and smoking, only depression symptoms and functional impairment showed significant associations. No significant genetic correlations were observed with anxiety disorder or schizophrenia. Comparisons using a block jackknife method revealed that the genetic correlations with external phenotypes did not significantly differ between impairment and symptom measures, except in the case of education. Here, the correlation was significantly weaker for impairment than for symptoms (*p* = 7.7 × 10^−4^ for depression symptoms and 1.0 × 10^−2^ for anxiety symptoms).

## Discussion

This study investigated the genetic influences on, and correlations between, self-reported functional impairment (WSAS) and current symptoms of depression (PHQ-9) and anxiety (GAD-7) in 17,130 individuals with lifetime depression or anxiety. We observed significant SNP-based heritability estimates for all three measures as well as genetic correlations among them. These findings indicate that functional impairment and symptom severity are each influenced by genetic variants that substantially overlap.

### Heritability

SNP-based heritability estimates for depression and anxiety symptoms were comparable to those reported for case–control definitions of MDD and anxiety disorder (e.g., 9% (Wray et al., 2018) and 26% (Purves et al., 2020), respectively). The heritability of functional impairment was similar to that of symptoms, aligning with prior twin-based estimates (Rijsdijk et al., 2003; Romeis et al., 2005).

### Phenotypic and genetic correlations between traits

Phenotypic correlations were consistent in size and pattern with previous estimates (Kroenke et al., 2001; Spitzer et al., 2006; Zahra et al., 2014) and existing evidence that symptom severity and functional impairment are partially independent. The stronger phenotypic correlation between functional impairment and depression symptoms, compared to anxiety symptoms, may reflect a greater functional impact of depression or conceptual overlap between the PHQ-9 and WSAS. Notably, although sleep difficulties, low energy, and impaired concentration feature in diagnostic criteria for both MDD and generalized anxiety disorder, they are included in the PHQ-9 but not the GAD-7. These symptoms appear to be especially relevant to functional impairment (Fried & Nesse, 2014), which may potentially drive the higher observed correlation. Further insights into the relationships between these measures could be gained by investigating item-level associations using factor or network analysis. For example, a factor analysis of PHQ-9 and GAD-7 items identified four factors (Thompson et al., 2021); how these relate to, or are changed by, the addition of WSAS items might reveal clinically useful presentations.

Genetic correlations between measures were higher than expected based on the corresponding phenotypic correlations, indicating substantial overlap in the common genetic variants associated with self-reported depression or anxiety symptom severity and functional impairment. These genetic correlations accounted for approximately one-fifth of the phenotypic correlations, which is likely to be lower than what a twin study capturing all genetic influences would estimate. The high genetic correlations suggest that much of the common genetic variant signal associated with self-reported functional impairment is also captured by symptom-based measures. This aligns with findings from the UK Biobank (Jermy et al., 2021) showing that adding components of a diagnostic questionnaire for depression, including a binary item assessing functional impairment, had little impact on heritability or relevant genetic correlations beyond the core symptoms. Together, these results suggest that it may not be crucial to supplement symptom-based scales with information on functional impairment for genetic variant discovery. Self-reported symptom scales also enable vastly larger sample sizes than are feasible with clinician-derived diagnostic instruments, which are essential for well-powered genetic analyses. Despite the value of data from symptom-based scales, they typically assess recent rather than lifetime symptoms. Evidence from the depression literature suggests that the PHQ-9 only captures a proportion of the genetic information relevant to diagnostic presentations, instead more closely reflecting a broader distress phenotype (Cai et al., 2020; Huang et al., 2023). From a clinical perspective, it is essential to assess functional impairment; it is relevant for diagnosis and treatment outcomes, prioritized by patients, and cannot be inferred from symptom scale scores alone.

Genetic correlations with external phenotypes were broadly similar in magnitude across all three measures (depression symptoms, anxiety symptoms, and functional impairment). The strongest correlations were positive with PTSD and negative with self-rated health. The high genetic correlation between PTSD and functional impairment is consistent with indications that PTSD is a particularly impairing condition (Olatunji et al. 2007). Comparisons between the genetic correlations revealed that the negative association with years of education was significantly weaker for functional impairment than for symptoms. This suggests that the genetic influences on lower educational attainment may be more closely related to liability to internalizing symptoms than to their functional consequences. Several correlations with depression and anxiety symptoms were similar to those reported from studies of case–control MDD and anxiety (Harris et al., 2017; Purves et al., 2020; Stein et al., 2021; Wray et al., 2018). On the other hand, unexpectedly weak or null genetic correlations were observed with case–control anxiety disorders, schizophrenia, anorexia, and MDD, as well as with neuroticism. These discrepancies between our analyses and the literature are likely attributable to selection bias, which is discussed further in the limitations.

### Strengths and limitations

This is the first study to perform SNP-based genetic analyses of the relationship between depression and anxiety symptoms and functional impairment. It is also one of the few genetic studies of functional impairment, an outcome of considerable clinical relevance. The measures used are widely employed in both clinical and research settings and have been validated across a range of cultures and patient groups (e.g., Mughal et al., 2020). However, several limitations should be noted when interpreting these results.

First, our analyses were restricted to individuals with lifetime depression or anxiety from the GLAD Study, a sample characterized by high rates of depression recurrence, treatment receipt, and comorbidity (Davies et al., 2019). Given that depression and anxiety are influenced by both genetic and environmental risk factors, individuals who experience them will generally have higher levels of disorder-related genetic variants than unaffected controls. As such, although phenotypic scores were approximately normally distributed, the sample likely overrepresents individuals at the upper end of the genetic risk distribution. This restricted range of relevant genetic variation limits the generalizability of our findings to a broader population. It likely also underlies the unexpectedly weak genetic correlations we observed with several external phenotypes, including case–control GWAS of psychiatric conditions and population-based traits such as neuroticism, which capture a broader spectrum of genetic liability. The interpretation of the genetic correlations is further complicated by the low statistical power of the internal phenotypes, as indicated by heritability *z*-scores below the suggested threshold of 4 (Zheng et al., 2017). On the other hand, the high genetic correlation between depression and anxiety symptoms was consistent with analyses of the same measures in the UK Biobank, a population-based sample (Thorp et al., 2021). In addition, investigating associations between symptom severity and functional impairment arguably requires a sample with nonzero levels of these traits, as impairment only becomes relevant in the presence of symptoms. In the present study, symptom and functional impairment scores were relatively normally distributed, in contrast to population-based samples where floor effects are common and many participants typically score zero. This strong skew makes a linear model unsuitable, and the transformation of zero-inflated distributions for GWAS has been criticized (e.g. Beasley, Erickson, & Allison, 2009). As such, analyses of these measures in the UK Biobank have been constrained, for example, dichotomizing the GAD-7 to perform a case–control GWAS and thereby sacrificing quantitative information (Purves et al., 2020).

Second, two common limitations of GWAS also apply to this study: low statistical power to detect genome-wide significant associations after Bonferroni correction, and limited sample diversity, particularly with respect to sex, education, and ancestry. These issues restricted, respectively, our ability to further investigate the genetic correlations between the measures and the generalizability of our findings.

Third, although widely used in clinical and research settings, all three measures were self-reported. Therefore, the relationships between these measures may, in part, be driven by negative cognitive biases that are observed in individuals both with and without clinically relevant levels of mental health problems (Roiser, Elliott, & Sahakian, 2012). Indeed, prior research using objective measures of impairment reported lower phenotypic correlations with symptoms (Kroenke et al., 2001; Spitzer et al., 2006).

### Future directions

Future studies of functional impairment would benefit from using more comprehensive measures, for example, by incorporating items on self-care (e.g., washing), and avoiding questions that apply only to a subset of respondents (e.g., ability to work). Greater insight may also be gained from objective indicators of impairment, such as work absences. Impairment has been proposed as a transdiagnostic phenotype to maximize sample sizes across mental health conditions (McGrath et al., 2013) and may be relevant to the general genetic liability underlying these disorders (Caspi et al., 2014). Functional impairment could also offer an additional phenotyping method when other information, such as symptom data, is unavailable. Testing this will require investigations of the genetic influences on functional impairment across a range of mental health disorders. In addition, it is important to consider that genetic correlations can result from multiple mechanisms. A genetic variant can influence both traits or may affect one trait, which then impacts the other (van Rheenen et al., 2019), and correlations can arise from genetically similar subgroups. In this study, of the moderate phenotypic correlations between symptoms and impairment, a small proportion was attributable to the strong genetic correlations between them. A twin-based design would be required to determine whether the remaining phenotypic overlap reflects environmental contributions, measurement error, or genetic factors not captured by common SNPs.

## Conclusions

Functional impairment is often overlooked in clinical and research contexts despite its clinical importance and only moderate phenotypic correlation with symptom severity. In this analysis of individuals with lifetime depression or anxiety, we found high genetic correlations between functional impairment and symptoms. This suggests that genetic analyses of functional impairment did not capture many additional variants relevant to full diagnostic presentations beyond those identified through symptom scores.

## Supporting information

Skelton et al. supplementary material Skelton et al. supplementary material

## Data Availability

The GLAD Study data are available via a data request application to the NIHR BioResource (https://bioresource.nihr.ac.uk/using-our-bioresource/academic-and-clinical-researchers/apply-for-bioresource-data/). The data are not publicly available due to restrictions outlined in the study protocol and specified to participants during the consent process. A specific data freeze is available, including the variables used for the analyses described in this article; email gladstudy@kcl.ac.uk for details.

## Supplementary material

The supplementary material for this article can be found at http://doi.org/10.1017/S0033291725101037.

## Analytical code availability

The code for the analyses included in this article is available at https://github.com/megskelton/impairment_sympt_overlap.
