# Supplementary material for: Genetic overlap between functional impairment and depression and anxiety symptom severity: evidence from the GLAD Study
Source: Psychol Med. 2025 Aug 5;55:e224. doi: 10.1017/S0033291725101037 (PMC12341029; doi:10.1017/S0033291725101037)
Supplement: Skelton et al. supplementary material [file S0033291725101037sup001.docx]

# Supplementary Information 1. PHQ-9, GAD-7 and WSAS measures

**Patient Health Questionnaire 9-item version (PHQ-9)** [^1^](https://paperpile.com/c/GZBq6a/et4B)

#
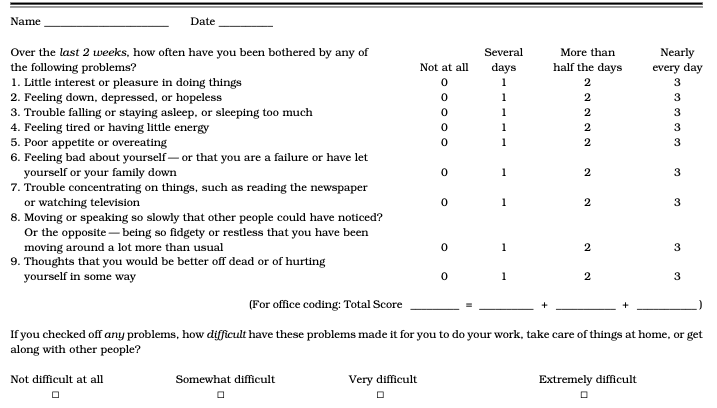
Generalised Anxiety Disorder 7-item scale (GAD-7) [^2^](https://paperpile.com/c/GZBq6a/rwZu)


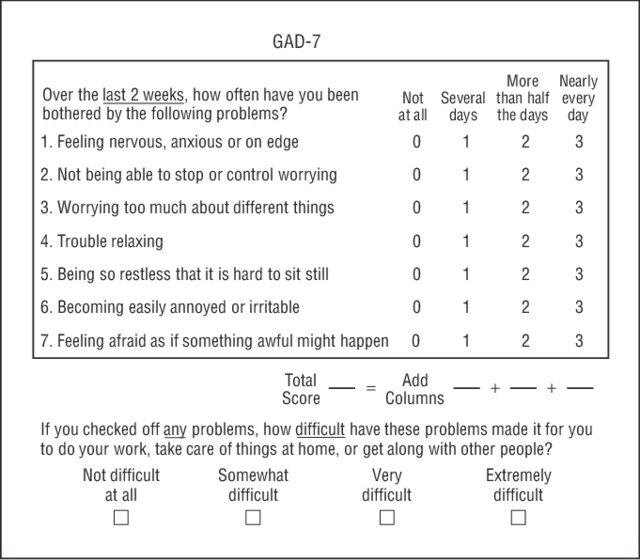


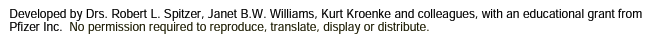


The papers describing the development of each of these measures [^1,2^](https://paperpile.com/c/GZBq6a/et4B+rwZu) detail a single functional impairment item that was presented alongside the nine and seven symptom items, to validate the total symptom scores. The item was, “If you checked off any problems, how difficult have these problems made it for you to do your work, take care of things at home, or get along with other people?”. Response options were: not difficult at all, somewhat difficult, very difficult, extremely difficult. In research and clinical settings (including NHS Talking Therapies services), depending on which of the freely available versions of the PHQ-9 and GAD-7 are used, this item may or may not be present. The item is not recorded in patient records within NHS Talking Therapies and was not presented to participants in the GLAD Study.

**Work and Social Adjustment Scale (WSAS)** [^3^](https://paperpile.com/c/GZBq6a/mWrP)

“Questions about your work and social life. People’s problems sometimes affect their ability to do certain day-to-day tasks in their lives. To rate your problems look at each section and determine on the scale provided how much your problem impairs your **current** ability to carry out the activity. Please complete this section thinking about what problem it was that led to you register for the Genetic Links to Anxiety and Depression Study. This includes but is not limited to anxiety, depression and sleep problems.”


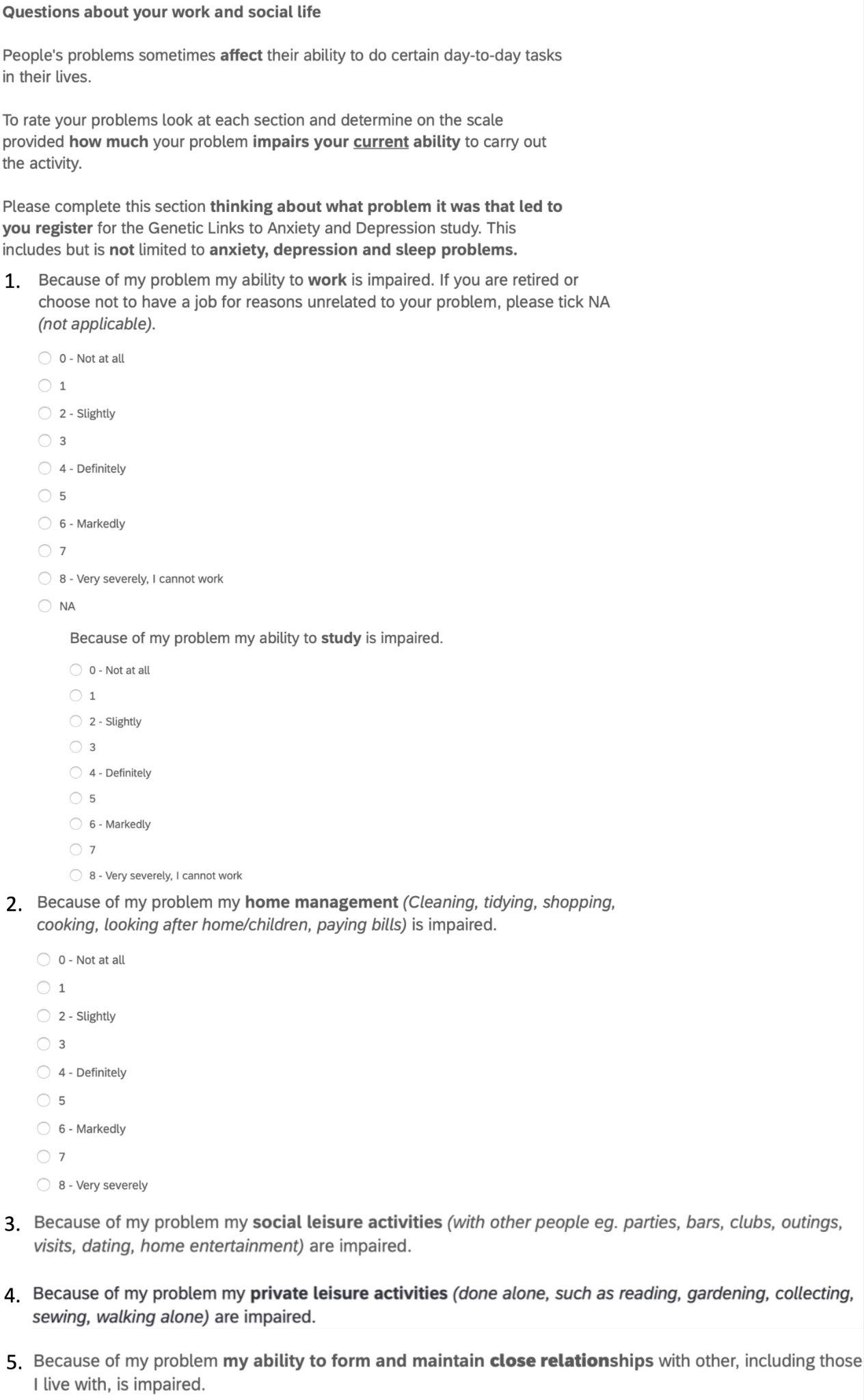


Within the GLAD Study, items (3), (4) and (5) had the same response options as item (2). Several months after the GLAD Study launched, an item on impairment in ability to study (shown above) was added to the sign-up questionnaire for individuals who selected ‘not applicable’ to the work item *and* endorsed student status. Work item NA responses were thereby replaced by valid study item responses, where present.

# Supplementary Information 2. Investigating the WSAS

*Phenotypic*

Of the 17,130 participants in the sample, 15,066 had a complete 5-item total WSAS score (‘WSAS-5-c’). There were 17,107 participants with a 4-item total score (i.e., all five items present or missing only one item). Of the 2,041 missing only one item, almost all (2,013) had responded ‘not applicable’ to the work item (and the study item, if applicable - see Supplementary Information 1).

Cronbach’s alpha for WSAS-5-c was 0.85 and for the 4-item score omitting the work item (‘WSAS-4-no-work’) was 0.83; internal reliability was marginally decreased without this item. In participants with all items complete, the phenotypic correlation between WSAS-4-no-work and the work item score was 0.66. The correlation between WSAS-5-c and WSAS-4-no-work was 0.98 but as the latter measure is the majority of the former it is not very informative; they will be highly similar within the same individual.

Comparisons between participants with WSAS-5-c scores and those with *only* WSAS-4-no-work scores are shown in Table *I*. The mean 4-item score in individuals missing the work item was significantly lower, as were PHQ9 and GAD7 scores. Participants with the work item missing were on average older than those with complete 5-item scores, and more often reported being retired or looking after the home or family than being employed.

Table *I*. Comparison of participants with complete data on the Work and Social Adjustment Scale (WSAS) and participants missing the work item of the WSAS

| **Variable** | **Full 5-item WSAS**  N = 15,066 | **Only 4-item WSAS; work missing**  N = 2,015 | ***p**** | **Effect size**** |
| --- | --- | --- | --- | --- |
| **Age** | 37.7 (13.0) | 52.8 (18.6) | < 0.001 | -0.94 |
| **Gender (Female)** | 11,793 (78%) | 1,531 (76%) | 0.019 | 0.02 |
| **Employment** |  |  | < 0.001 | 0.67 |
| In paid employment or self-employed | 10,183 (68%) | 102 (5%) |  |  |
| Full or part-time student | 1,813 (12%) | 245 (12%) |  |  |
| Unable to work because of sickness or disability | 1,618 (11%) | 237 (12%) |  |  |
| Retired | 213 (1%) | 1,013 (51%) |  |  |
| Unemployed | 528 (4%) | 81 (4%) |  |  |
| Looking after home and/or family | 322 (2%) | 230 (12%) |  |  |
| Doing unpaid or voluntary work | 253 (2%) | 62 (3%) |  |  |
| None of the above | 106 (1%) | 36 (2%) |  |  |
| **University degree (Yes)** | 8,855 (59%) | 908 (45%) | < 0.001 | 0.09 |
| **Depression symptoms PHQ9** | 11.4 (6.9) | 10.0 (6.9) | < 0.001 | 0.20 |
| N missing | 31 | 17 |  |  |
| **Anxiety symptoms GAD7** | 9.1 (5.9) | 7.6 (6.0) | < 0.001 | 0.26 |
| N missing | 23 | 7 |  |  |
| **Functional impairment WSAS 4-item** | 13.7 (7.4) | 11.3 (7.4) | < 0.001 | 0.32 |
| **CIDI-SF Depression (Case)** | 12,997 (89%) | 1,662 (88%) | 0.14 | 0.01 |
| **CIDI-SF Anxiety (Case)** | 8,288 (62%) | 946 (57%) | < 0.001 | 0.03 |
| Values are mean (SD) *or* n (%). * Welch’s two-sample t-test, or Pearson's Chi-squared test, or Fisher's exact test. ** Cohen’s D or Cramer’s V. CIDI-SF = Composite International Diagnostic Interview short-form, questionnaire-based. | | | | |

*Genetic*

We were unable to perform genetic comparisons using WSAS-4-no-work restricted to participants who responded ‘not applicable’ to the work item (to assess the feasibility of imputation from a genetic point of view) as this group was too small (N = 2,013); h^2^_SNP_ estimates were not significantly different from zero. The bivariate-GREML estimate of genetic correlation between WSAS-4-no-work (N = 17,080; h^2^_SNP_ 0.11 (SE = 0.03)) and the work item (N = 15,094; h^2^_SNP_ 0.07 (SE = 0.04)) was *r_g_* = 0.93 (SE = 0.12). The genetic correlation between WSAS-5-c (N = 15,065; h^2^_SNP_ 0.13 (SE = 0.04) and the work item was *r_g_* = 0.93 (SE = 0.13). These genetic correlations are unlikely to be significantly different from 1 and therefore, despite the phenotypic differences described above and the possibility that the missingness was not at random, we made the decision to use an individual mean imputed 5-item WSAS (N = 17,107) for the main analysis. The sample is larger than that for WSAS-4-no-work as it includes participants missing *any* one item, not only the work item.

# Supplementary Information 3. Genetic quality control

Genetic ancestry was inferred using principal component analysis (PCA), anchoring participants against reference populations from the 1000 Genomes Project and Gujarati Indian HapMap dataset. In PLINK^4^, identity-by-state (IBS) values were computed using the ‘*genome*’ function, followed by complete linkage clustering with the ‘*cluster*’ option to identify population substructure. Genetic ancestry groups were then assigned based on visual inspection of PCA plots and empirically derived thresholds on PC axes to capture clusters. These thresholds were determined based on clustering patterns with the reference samples. Individuals were assigned to European ancestry if their PC1 value was greater than −0.0035, while other ancestry groups (South Asian, East Asian, African) were defined using empirically derived cutoffs across multiple PCs (e.g., PC1 < −0.0250 and PC2 < −0.02 for East Asian).

Genetic variants were excluded if: missing > 2%, minor allele frequency < 1%, or Hardy-Weinberg equilibrium *p* < 1x10^-8^. Participants were excluded if: missing data for > 5% of variants or did not cluster within the European group of the PCA, due to limited sample sizes in other ancestry groups. Identity by descent (IBD) was inferred from IBS computed in PLINK. We calculated the average pi-hat value for each individual across all pairwise comparisons and those > 3 standard deviations above the cohort mean were excluded (n = 302). These outliers had globally elevated relatedness meaning they were more closely related to many more individuals in the cohort than expected, with patterns unlikely to result from cryptic relatedness. Such patterns could arise due to DNA sample contamination. As a precaution, we excluded these individuals from the analysis. We did not exclude other relatives as our downstream methods handle relatedness or filter where necessary. Sex mismatches, which can be indicative of genotyping errors or contamination, were also excluded (n = 58). Mismatches were defined as discordance between genetically inferred sex (F-statistic males > 0.8, females < 0.2) and reported sex assigned at birth, alongside intermediate X chromosome F-statistic values (0.2 - 0.8). Genotype phasing was performed using Eagle (Version 2.4), and imputation was carried out with Minimac4 via the TOPMed reference panel (Version r2, GRCh38 ^5-7^). Alongside the aforementioned quality control thresholds, we filtered to imputation quality (R^2^) > 0.3. Following quality control there were 564,245 genotyped and 7,027,957 imputed variants.

# Supplementary Table 1. Description of external phenotypes for genetic comparison

| **Phenotype** | **Author & PMID** | **N**  **(n cases)** | **Obs h^2^_SNP_** | **Obs h^2^_SNP_ SE** | **z-score** |
| --- | --- | --- | --- | --- | --- |
| Major depression (Mega-analysis, excluding 23andme & UK Biobank†: Case-control status derived from structured clinical interview, inpatient medical records) | Wray et al. (2018) 29700475 | 143,265 (45,591) | 0.069 | 0.005 | 14.06 |
| Anxiety (UK Biobank: Case-control status derived from algorithm of CIDI-SF questionnaire, self-report diagnosis) | Purves et al. (2020)  31748690 | 83,566 (25,453) | 0.103 | 0.007 | 14.90 |
| Schizophrenia (Meta-analysis: Case-control status derived from clinical diagnosis. European subsample) | Trubetskoy et al. (2022) 35396580 | 130,644 (53,386) | 0.361 | 0.013 | 27.11 |
| Attention deficit and hyperactivity disorder (ADHD, meta-analysis: Case-control status derived from clinical diagnosis) | Demontis et al. (2023) 36702997 | 225,534 (38,691) | 0.093 | 0.004 | 22.59 |
| Post-traumatic stress disorder (PTSD, Million Veteran Program: Case-control status derived from electronic health record algorithm) | Stein et al. (2021) 33510476 | 214,408 (36,301) | 0.035 | 0.003 | 10.64 |
| Neuroticism (Meta-analysis: Score on a personality inventory) | Gupta et al. (2024) 39134740 | 623,482 | 0.077 | 0.003 | 27.46 |
| Tiredness (UK Biobank: Score from single item on recent frequency of tiredness or little energy) | Deary et al. (2018) 28322280 | 108,976 | 0.063 | 0.006 | 9.98 |
| Years of education (Meta-analysis: Derived from survey measures of highest educational qualification) | Lee et al. (2018)  30038396 | 766,345 | 0.109 | 0.003 | 35.00 |
| Smoking (Meta-analysis excluding 23andme: Ever regular smoker versus never regular smoker item) | Liu et al. (2019) 30643251 | 632,802 (311,629)* | 0.066 | 0.003 | 25.54 |
| Self-rated health  (UK Biobank: Single item score) | Harris et al. (2017)  27864402 | 111,483 | 0.088 | 0.006 | 14.38 |

For all summary statistics: mean chi-square of the test statistic > 1.0, intercept within 0.9-1.1 range, heritability *z*-score > 4 (as recommended in ref [^8^](https://paperpile.com/c/GZBq6a/SlMdm)). Heritability estimates are from the genetic correlation with PHQ9 rather than the cited analyses and could differ due to the restriction to variants shared across samples. ^†^ This exclusion ensures as close an approximation of clinical major depression as possible, as UK Biobank and 23andme assessments primarily relied on self-reported diagnosis or core symptoms combined with help-seeking. *Estimated from their supplementary materials.

#
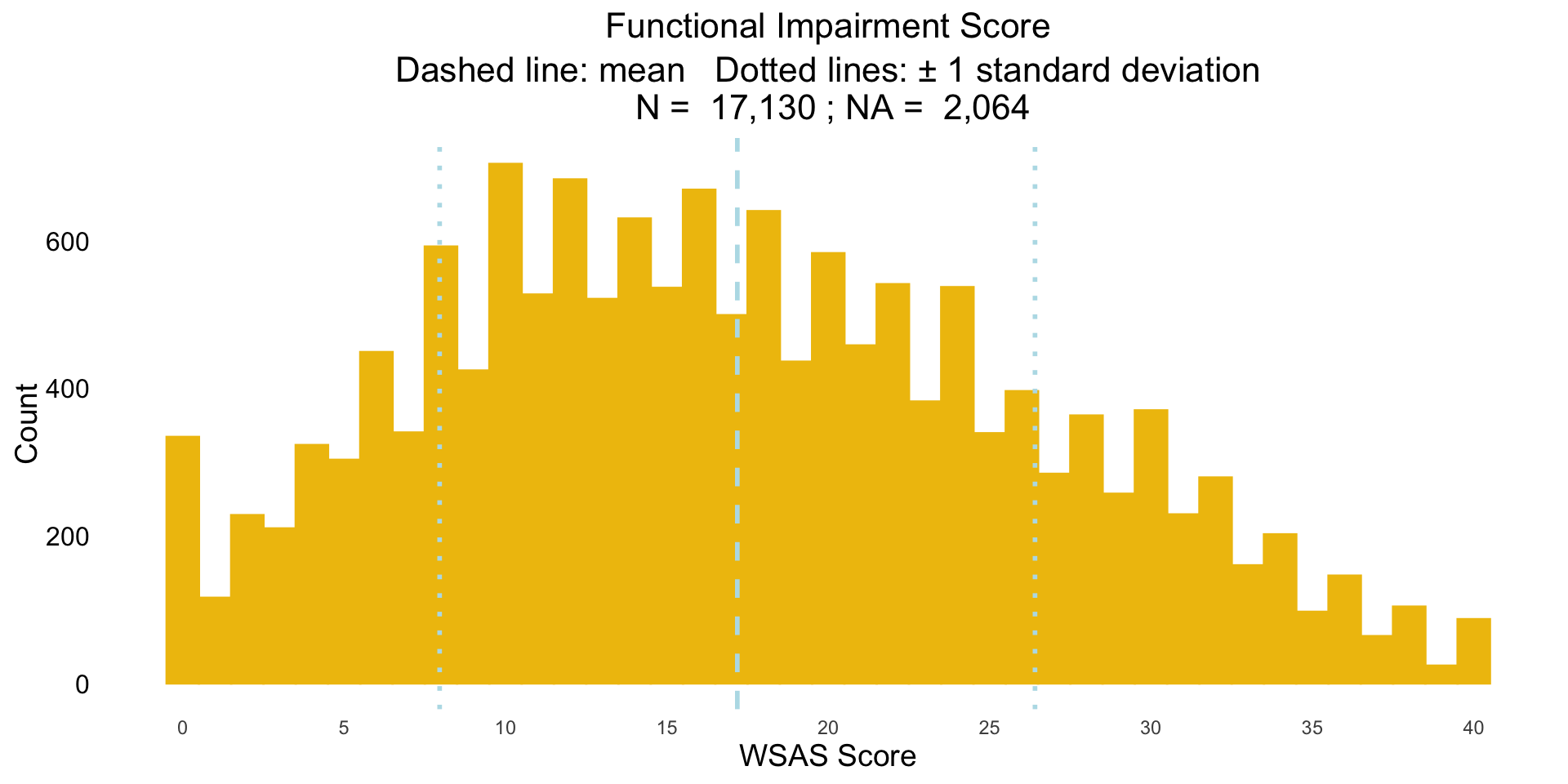
Supplementary Figure 1. Histograms showing the distribution of each phenotype
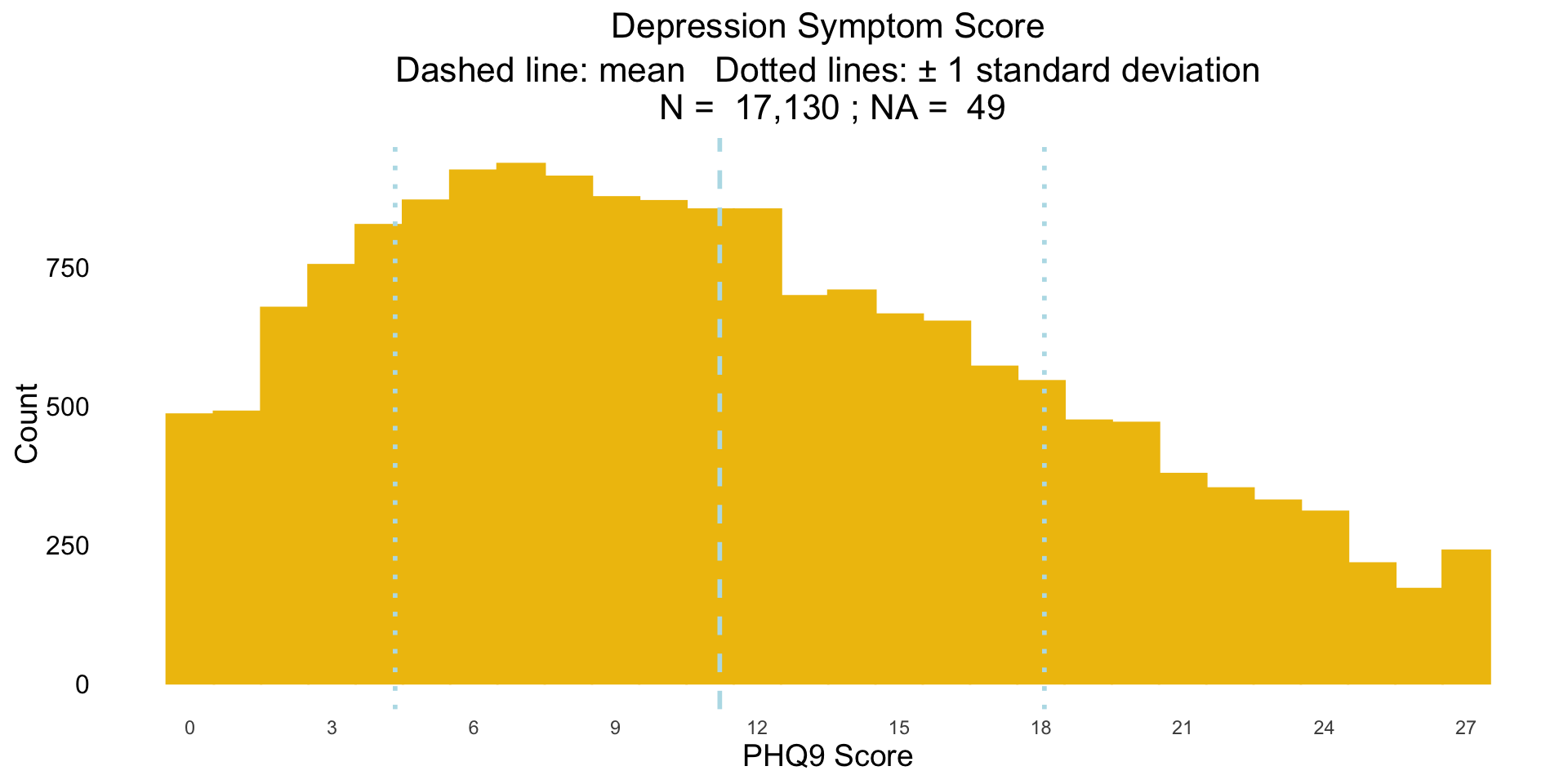


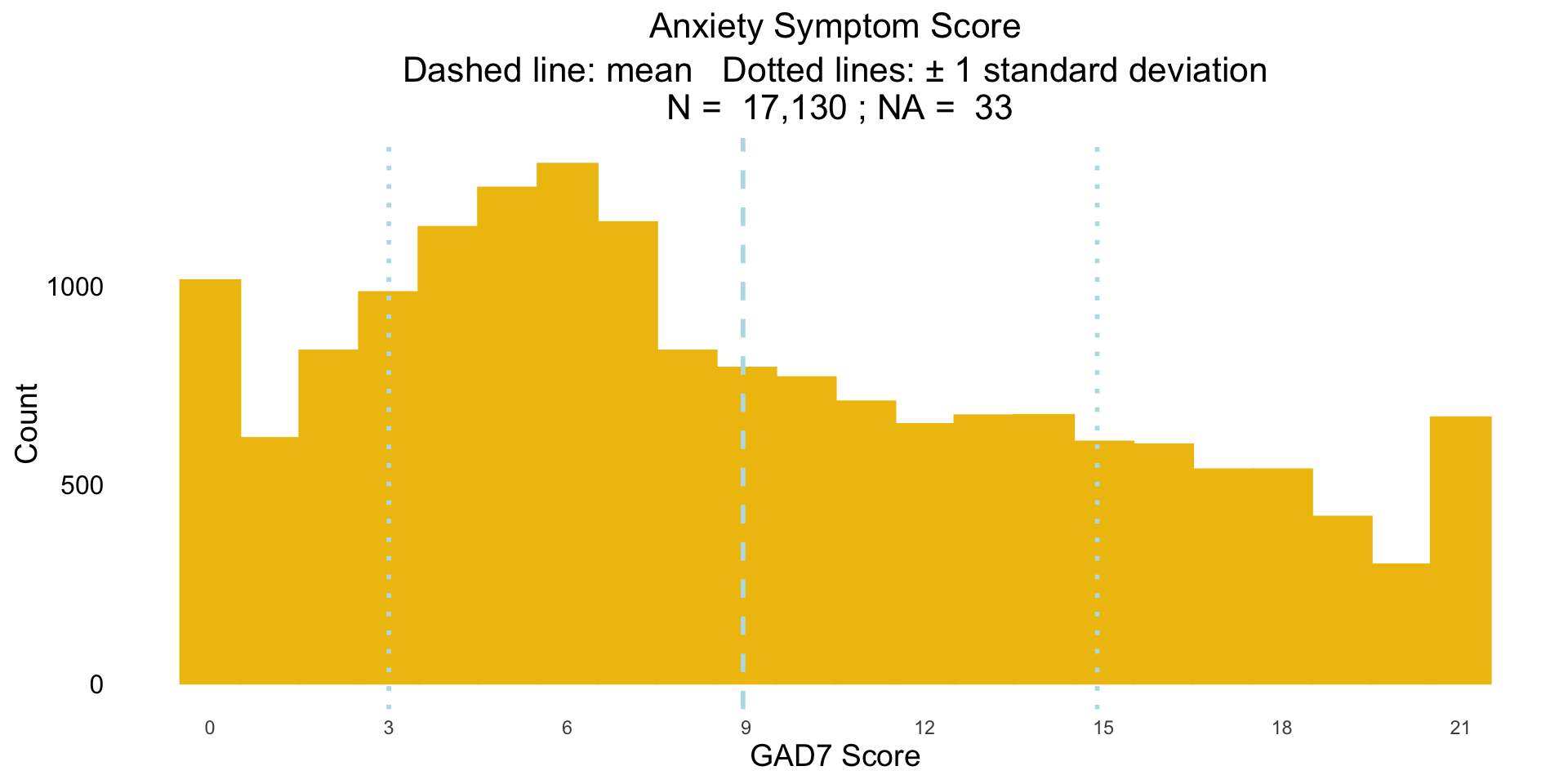
PHQ9 = depression symptoms (top left), GAD7 = anxiety symptoms (top right), WSAS = functional impairment (bottom).

Skewness values were below |1|, the threshold typically used to identify excessive skewness, and mean and median values were similar (PHQ-9: skewness = 0.34, mean = 11.2, median = 10.0; GAD-7: skewness = 0.39, mean = 8.85, median = 8.0. WSAS: skewness = 0.25, mean = 17.18, median = 16.0).

# Supplementary Figure 2. Manhattan and quantile-quantile (QQ) plots from the genome-wide association study (GWAS) of each phenotype

Depression symptoms (PHQ9)


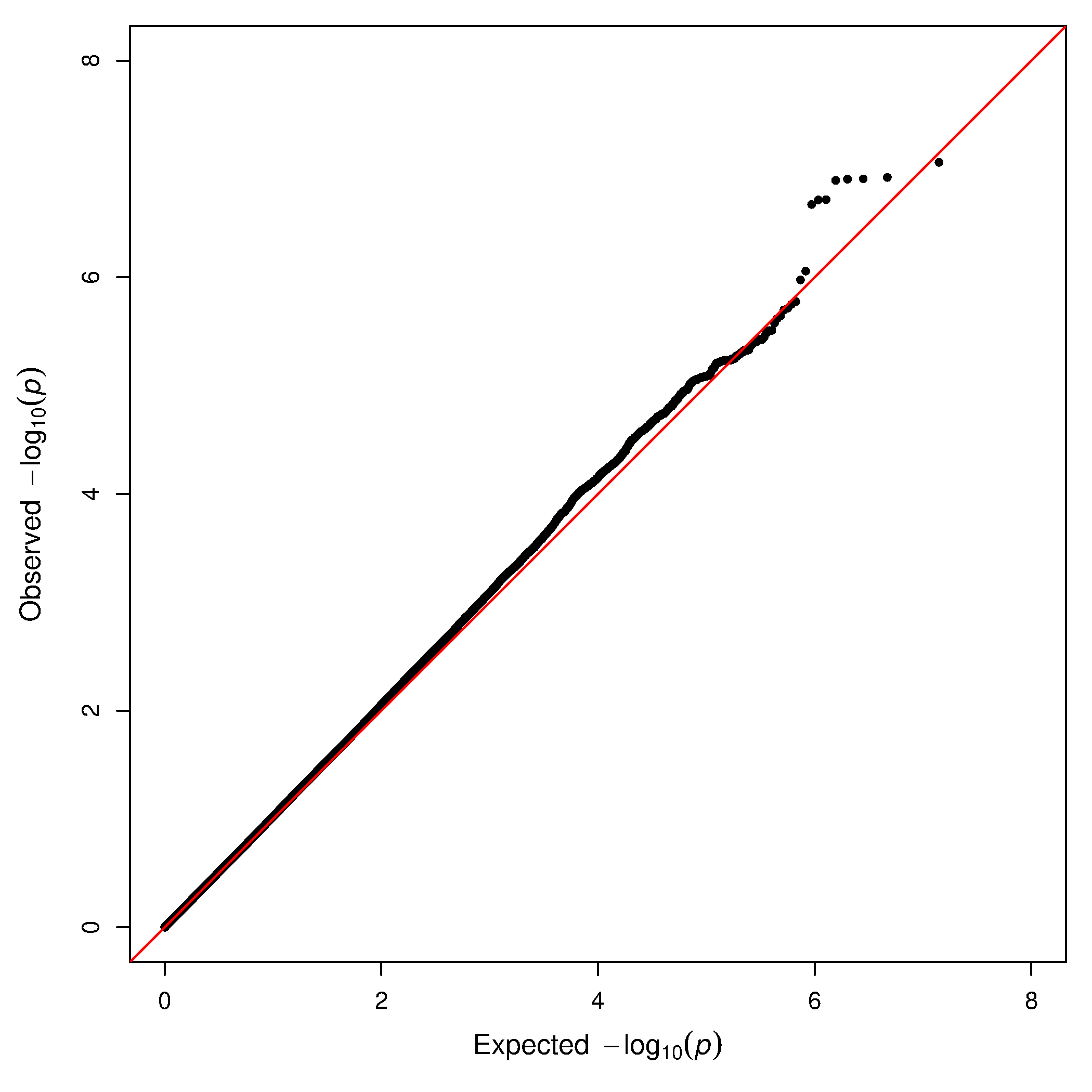


λ = 1.047


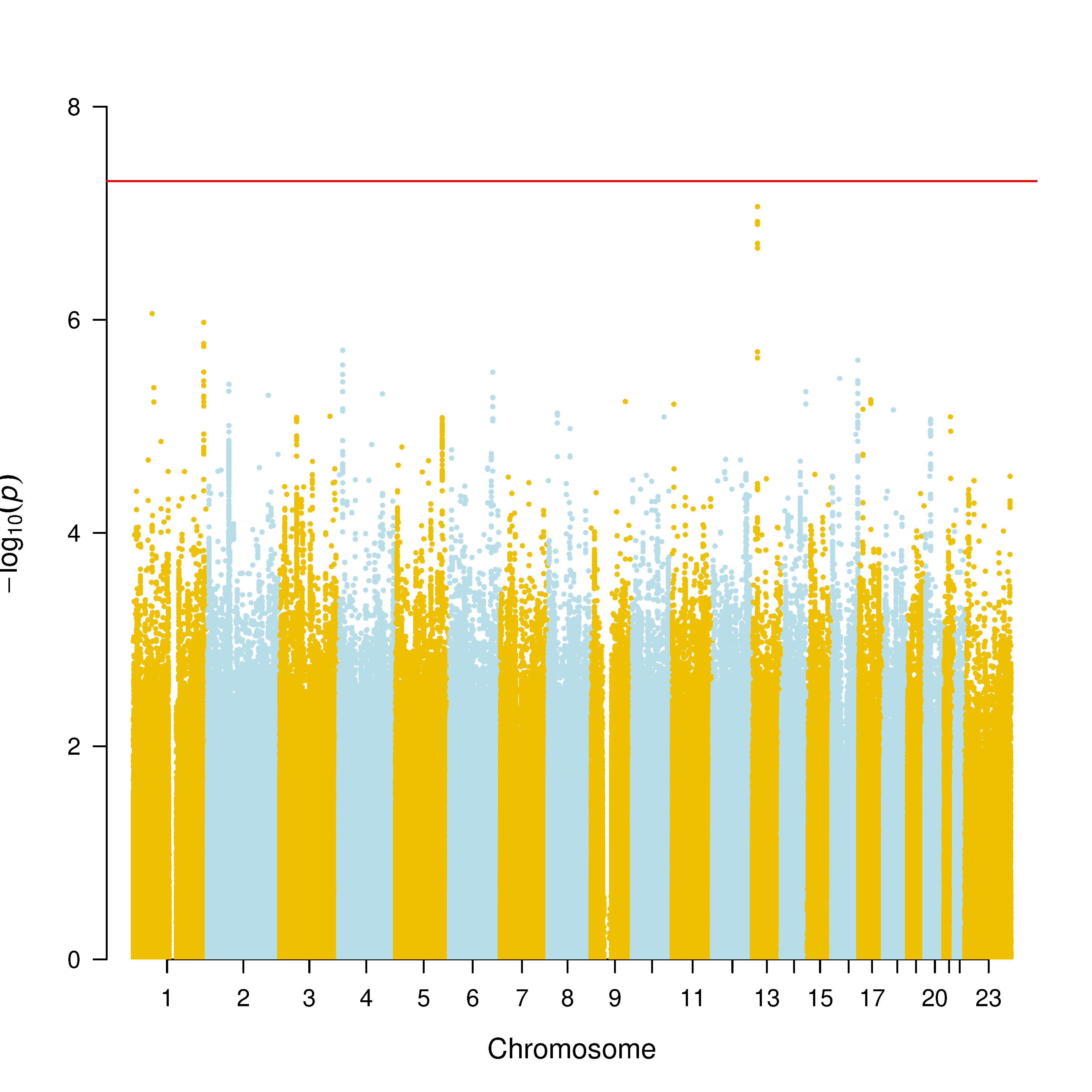


Anxiety symptoms (GAD7)


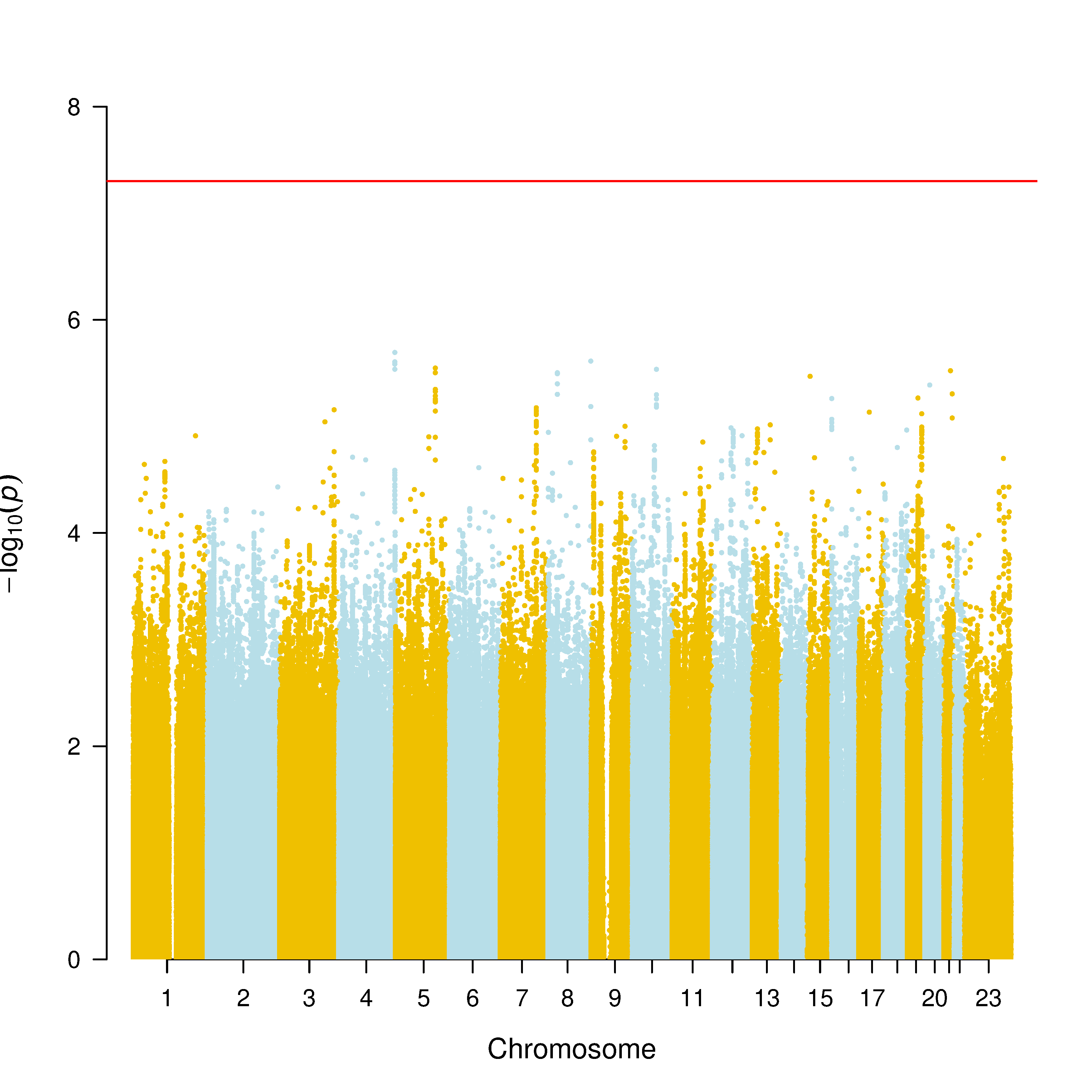


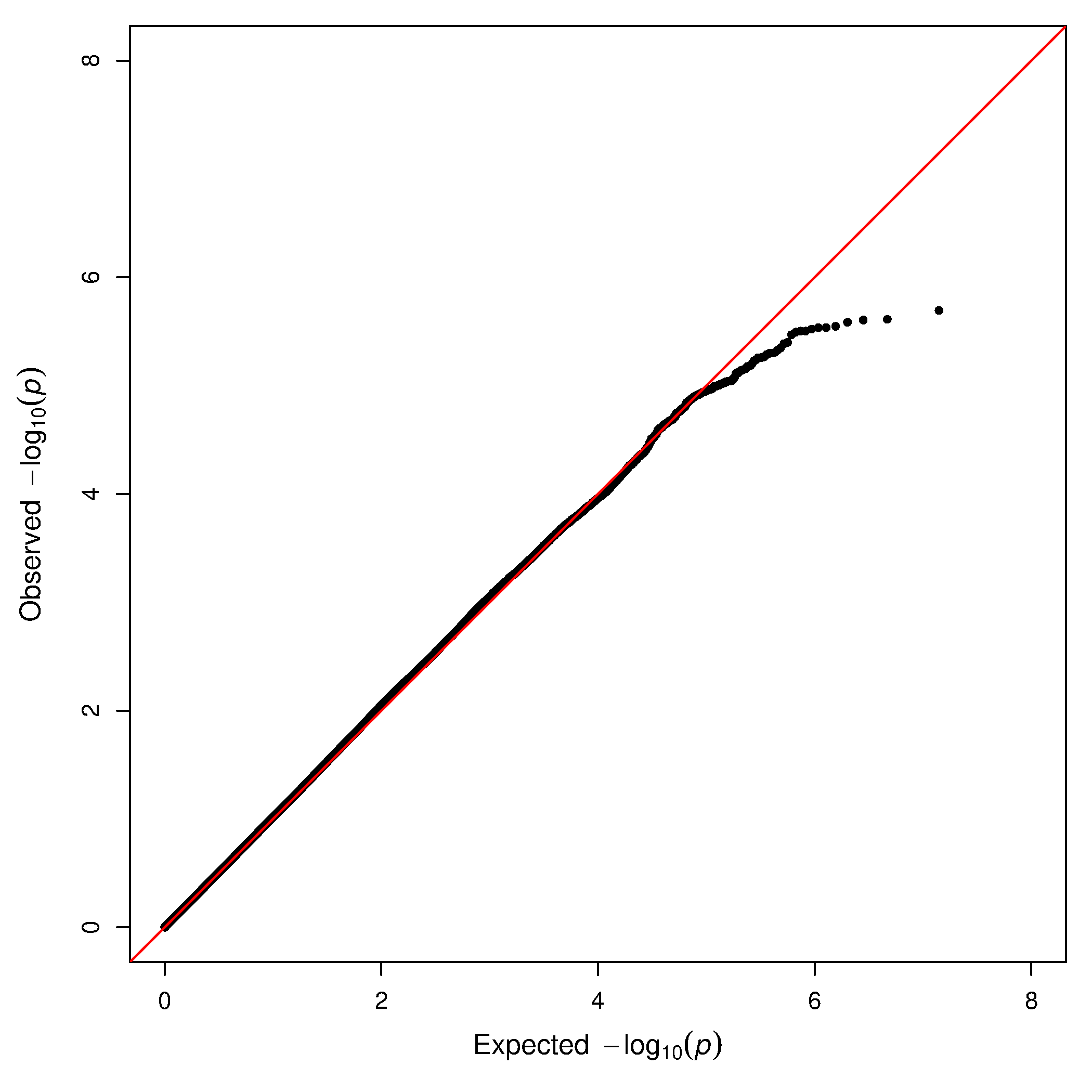


λ = 1.028

Functional impairment (WSAS)

**
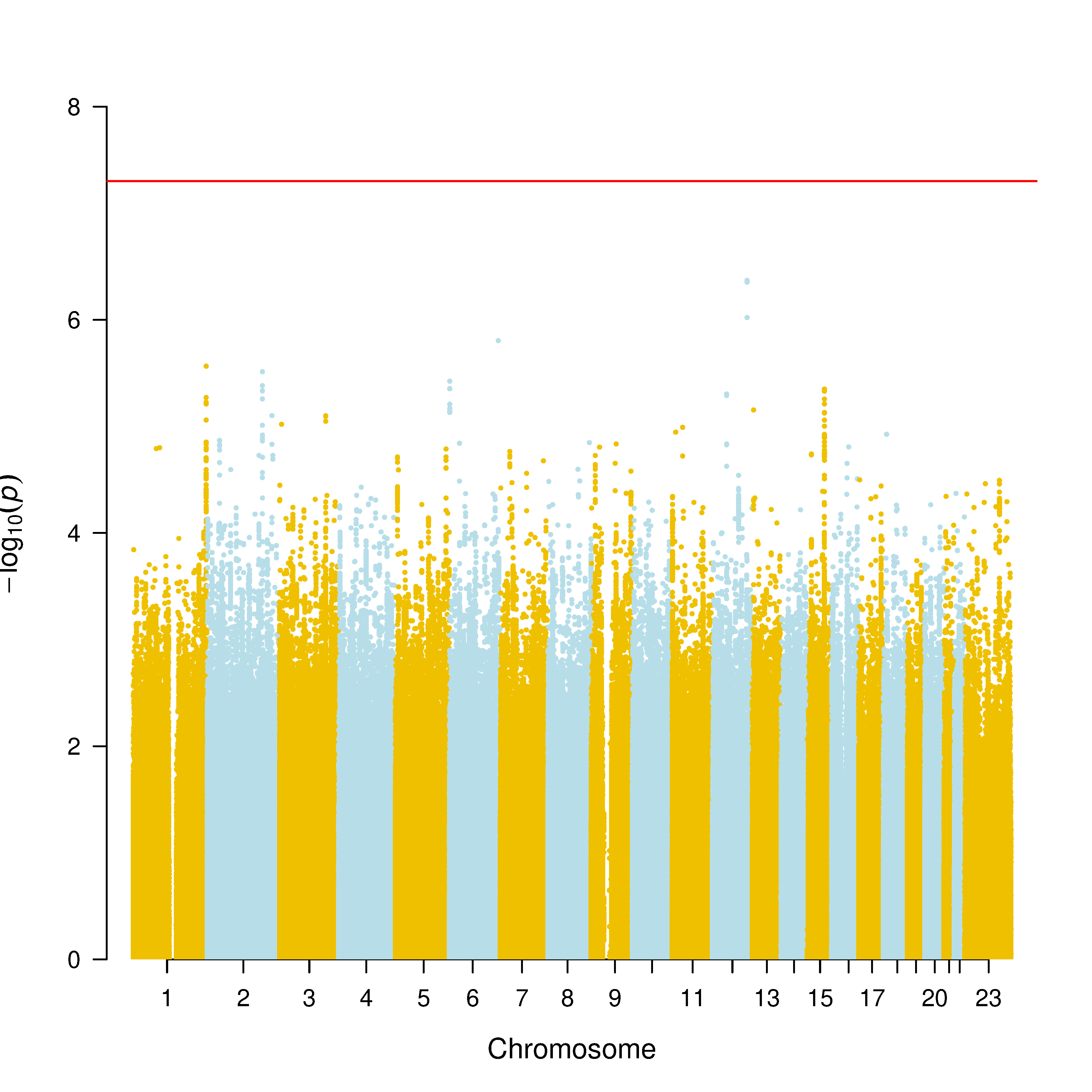
**


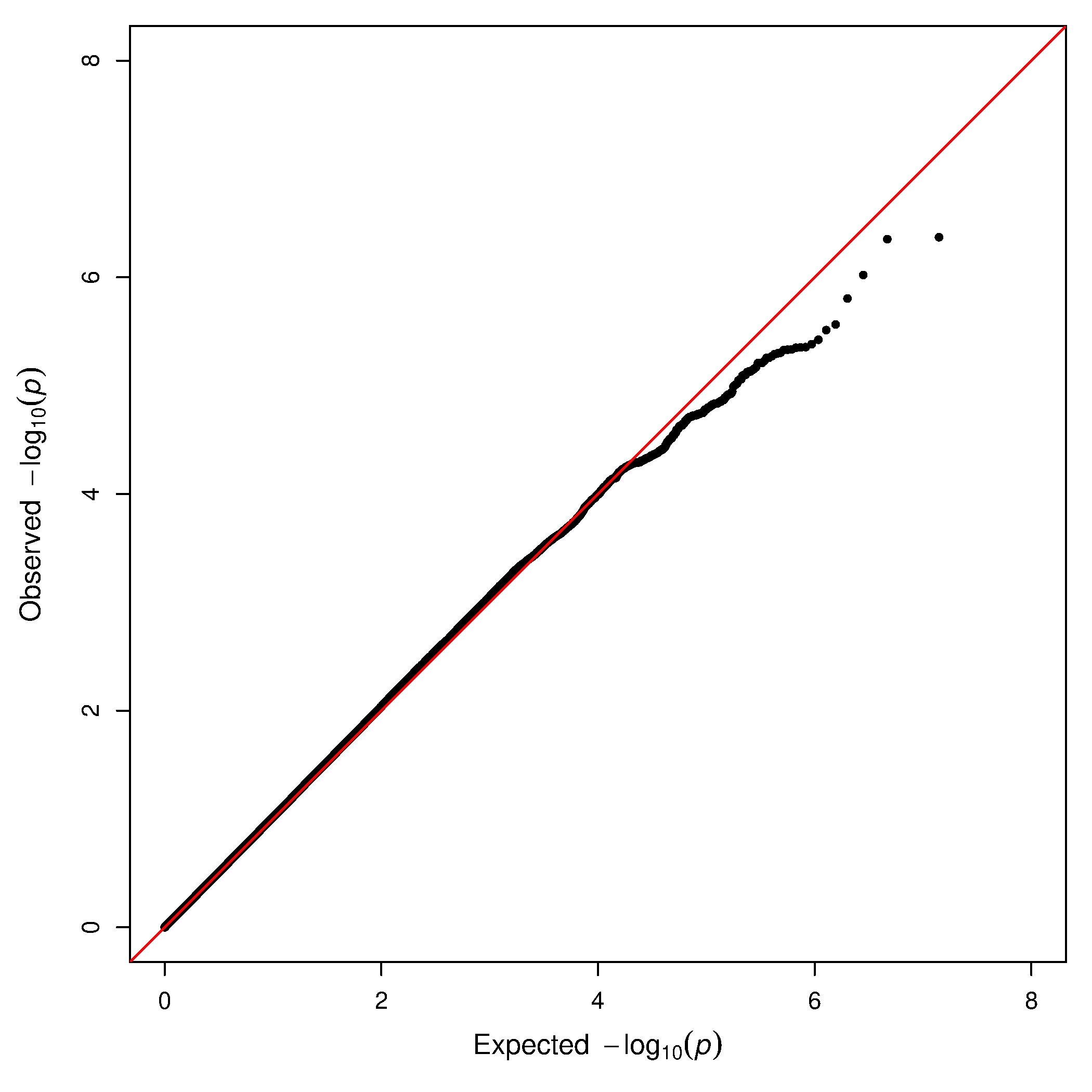


λ = 1.032

Manhattan plot red line = genome-wide significance threshold, corrected for multiple testing (*p* < 5x10^-8^); points above this line represent variants significantly associated with the phenotype. QQ plot red line = null hypothesis of no significantly associated variants. A tail of points above this line in the top right would represent deviations from the null indicating true genetic signal.

# Supplementary Table 2. Phenotypic correlations between symptoms and functional impairment

|  | **r_p_** | **95% CI** | ***p (H_0_ = 0)*** | ***p (H_0_ = 1)*** |
| --- | --- | --- | --- | --- |
| PHQ9-GAD7 | 0.69 | 0.69 - 0.70 | 8.8 x 10^-228^ * | <2.2 x 10^-308 †^ |
| PHQ9-WSAS | 0.63 | 0.62 - 0.64 | <2.2 x 10^-308 †^ * | <2.2 x 10^-308 †^ |
| GAD7-WSAS | 0.50 | 0.48 - 0.51 | <2.2 x 10^-308 †^ * | <2.2 x 10^-308 †^ |

PHQ9 = depression symptoms, GAD7 = anxiety symptoms, WSAS = functional impairment. 95% CI = 95% confidence interval. *p (H_0_ = 0)* and *(H_0_ = 1)* = significance test for null hypothesis of a correlation of 0 or 1, respectively. * Significant at *p* < 0.017 (Bonferroni correction for 3 tests). ^†^ Exact p-values exceeded the limit of R’s floating-point precision (<2.2 x 10^-308^).

# Supplementary Table 3. Bivariate-GREML Genetic correlation (*r_g_*) estimates between symptoms and functional impairment

|  | **r_g_** | **95% CI** | ***p (H_0_ = 0)*** | ***p (H_0_ = 1)*** |
| --- | --- | --- | --- | --- |
| PHQ9-GAD7 | 0.86 | 0.75 - 0.98 | 3.3x10^-8^ * | 6.8x10^-3^ |
| PHQ9-WSAS | 0.87 | 0.70 - 1.05 | 1.5x10^-6^ * | 0.098 |
| GAD7-WSAS | 0.79 | 0.57 - 1.02 | 1.3x10^-5^ * | 0.049 |

PHQ9 = depression symptoms, GAD7 = anxiety symptoms, WSAS = functional impairment. 95% CI = 95% confidence interval, calculated as *r_g_* +/- (1.96*SE). *p (H_0_ = 0)* and *(H_0_ = 1)* = significance test for null hypothesis of a correlation of 0 or 1, respectively. * Significant at *p* < 0.017 (Bonferroni correction for 3 tests).

# Supplementary Information 4. Analysis of complete case WSAS score and 4-item WSAS score omitting the work item, including LDSC heritability and genetic correlation estimates

## Figure *I*. Manhattan and quantile-quantile (QQ) plots from the genome-wide association study of each phenotype

Complete case WSAS score (WSAS-5-c; N = 15,065)


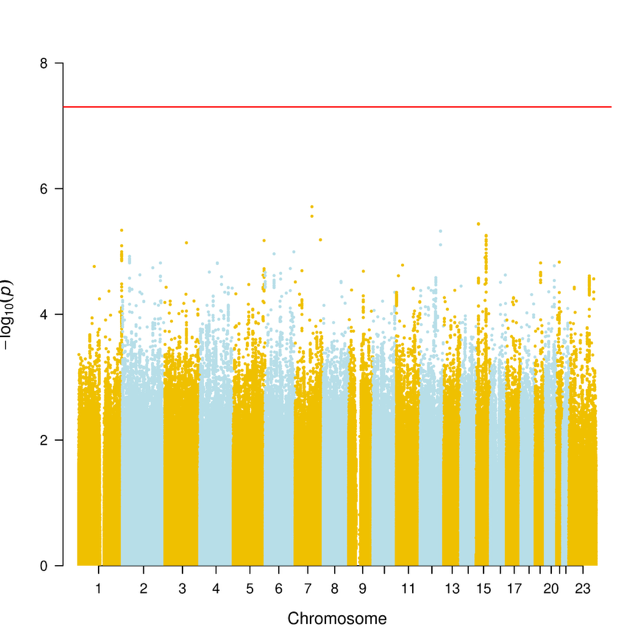


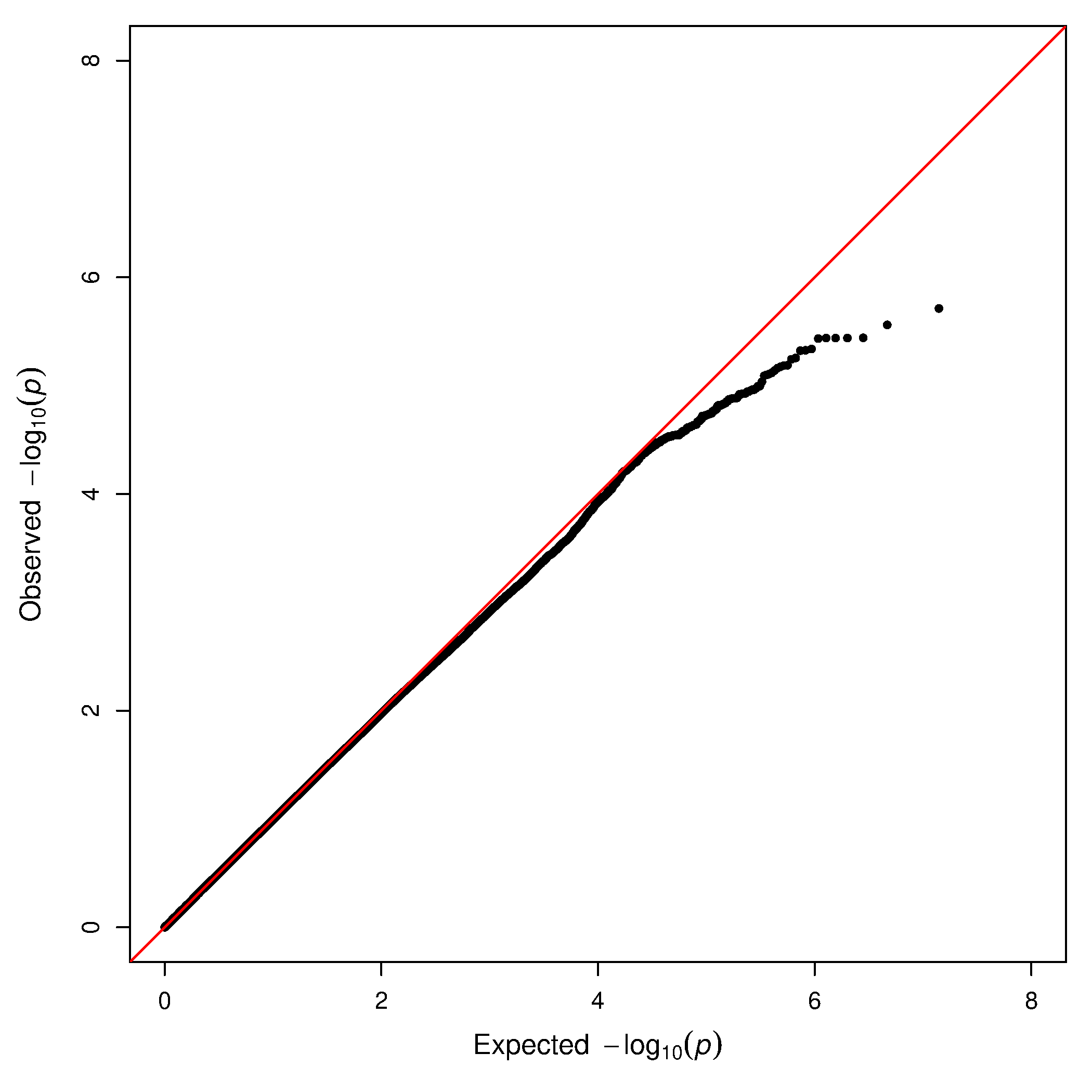


λ = 1.005

4-item WSAS score omitting the work item (WSAS-4-no-work; N = 17,080)


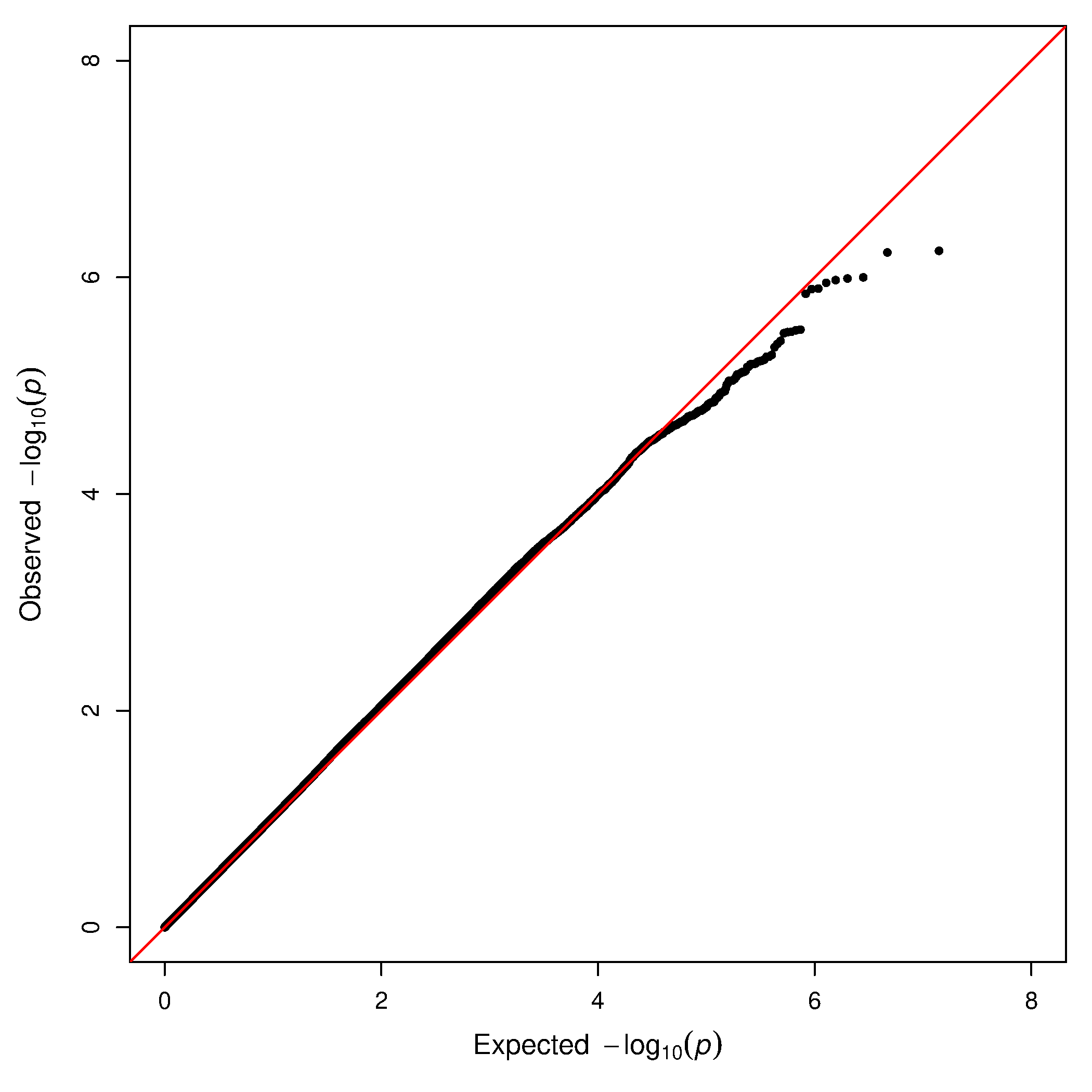


λ = 1.034

### **
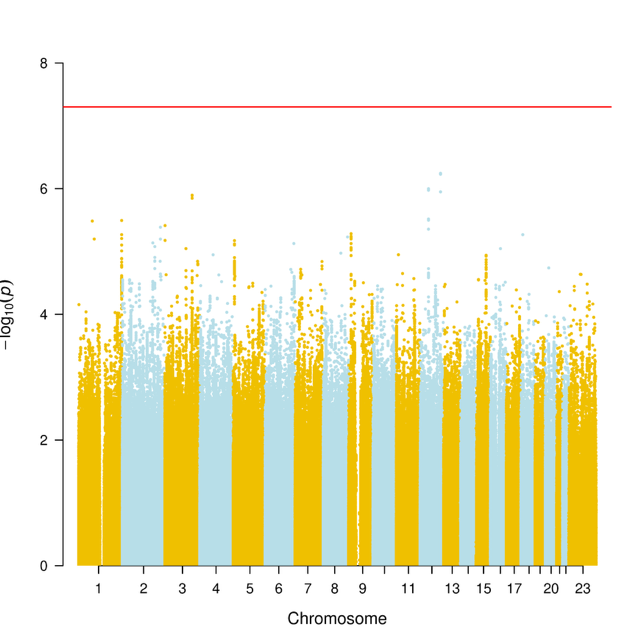
**

The red line on the Manhattan plot indicates the genome-wide significance threshold, corrected for multiple testing (p < 5x10^-8^); points above this line represent variants significantly associated with the phenotype. The red line on the QQ plot presents the null hypothesis of no significantly associated variants. A tail of points above this line in the top right of the plot would represent deviations from the null indicating true genetic signal.

## Table *I*. GCTA-GREML SNP-based heritability estimates

| **Trait** | **h^2^** | **h^2^ SE** | **95% CI** | **z** | ***p*** | **n *** |
| --- | --- | --- | --- | --- | --- | --- |
| **PHQ9** | 0.192 | 0.035 | 0.123 - 0.260 | 5.486 | 6x10^-9^ | 16709 |
| **GAD7** | 0.173 | 0.035 | 0.104 - 0.241 | 4.943 | 2x10^-7^ | 16725 |
| **WSAS** | 0.111 | 0.033 | 0.045 - 0.176 | 3.364 | 2x10^-4^ | 16736 |
| **WSAS-5-c** | 0.133 | 0.038 | 0.059 - 0.208 | 3.500 | 1x10^-4^ | 14744 |
| **WSAS-4-nw** | 0.111 | 0.033 | 0.045 - 0.176 | 3.364 | 2x10^-4^ | 16710 |

PHQ9 = depression symptoms, GAD7 = anxiety symptoms, WSAS = imputed 5-item functional impairment score used in main analysis, WSAS-5-c = complete case 5-item functional impairment score, WSAS-4-nw = 4-item functional impairment score omitting the work item. h^2^ = heritability, SE = standard error, 95% CI = 95% confidence interval calculated as h^2^+/- (1.96*SE),* Influenced by pre-processing for GCTA-GREML i.e., removal of close relatives.

## Table *II*. LDSC SNP-based heritability estimates

LDSC uses summary statistics and an external reference panel for LD scores, and therefore typically provides lower estimates than GCTA-GREML, which uses individual-level genotype data ^9,10^.

| **Trait** | **h^2^** | **h^2^ SE** | **95% CI** | ***p**** | ***z*** | **λ** | **Mean χ^2^** | **Int.** | **Int. SE** |
| --- | --- | --- | --- | --- | --- | --- | --- | --- | --- |
| **PHQ9** | 0.104 | 0.029 | 0.048 - 0.160 | 3x10^-4^ | 3.660 | 1.047 | 1.046 | 1.010 | 0.007 |
| **GAD7** | 0.082 | 0.030 | 0.024 - 0.141 | 0.006 | 2.774 | 1.028 | 1.034 | 1.007 | 0.007 |
| **WSAS** | 0.117 | 0.031 | 0.056 - 0.177 | 2x10^-4^ | 3.761 | 1.032 | 1.033 | 0.993 | 0.007 |
| **WSAS-5-c** | 0.125 | 0.032 | 0.062 - 0.188 | 1x10^-4^ | 3.873 | 1.005 | 1.001 | 0.963 | 0.007 |
| **WSAS-4-nw** | 0.111 | 0.030 | 0.053 - 0.170 | 2x10^-4^ | 3.735 | 1.034 | 1.034 | 0.995 | 0.007 |

PHQ9 = depression symptoms, GAD7 = anxiety symptoms, WSAS = imputed 5-item functional impairment score used in main analysis, WSAS-5-c = complete case 5-item functional impairment score, WSAS-4-nw = 4-item functional impairment score omitting the work item. h^2^ = heritability, SE = standard error, 95% CI = 95% confidence interval calculated as h^2^+/- (1.96*SE), Int = LDSC intercept. *Calculated in R as pchisq((h^2^/se)^2,1,F).

## Table *III*. Bivariate-GREML genetic correlation (*r_g_*) estimates

|  | ***r_g_*** | **95% CI** | ***p*** | ***z*** |
| --- | --- | --- | --- | --- |
| **PHQ9 – GAD7** | 0.86 | 0.75 - 0.98 | 3.3x10^-8^ | 15.07 |
| **PHQ9 – WSAS** | 0.87 | 0.70 - 1.05 | 1.5x10^-6^ | 10.0 |
| **GAD7 – WSAS** | 0.79 | 0.57 - 1.02 | 1.3x10^-5^ | 6.82 |
| **PHQ9 – WSAS-5-c** | 0.84 | 0.66 - 1.01 | 1.6x10^-6^ | 9.43 |
| **PHQ9 – WSAS-4-nw** | 0.90 | 0.73 - 1.07 | 6.4x10^-7^ | 10.27 |
| **GAD7 – WSAS-5-c** | 0.73 | 0.50 - 0.95 | 2.9x10^-5^ | 6.29 |
| **GAD7 – WSAS-4-nw** | 0.78 | 0.54 - 1.01 | 1.8x10^-5^ | 6.55 |
| **WSAS-5-c – WSAS-4-nw** | 1 | 0.99 - 1.01 | <1x10^-300†^ | 166.90 |

PHQ9 = depression symptoms, GAD7 = anxiety symptoms, WSAS = imputed 5-item functional impairment score used in main analysis, WSAS-5-c = complete case 5-item functional impairment score, WSAS-4-nw = 4-item functional impairment score omitting the work item. 95% CI = 95% confidence interval calculated as *r_g_* +/- (1.96*SE). ^†^ Value returned from GCTA 0.0000x10^00^ indicated the true *p* was lower than the machine precision threshold.

## Table *IV*. LDSC genetic correlation (*r_g_*) estimates

|  | ***r_g_*** | **95% CI** | ***p*** | ***z*** |
| --- | --- | --- | --- | --- |
| **PHQ9 - GAD7** | 0.85 | 0.63 - 1.07 | 5x10^-14^ | 7.52 |
| **PHQ9 - WSAS** | 0.82 | 0.61 - 1.03 | 1x10^-14^ | 7.70 |
| **GAD7 - WSAS** | 0.77 | 0.46 - 1.08 | 1x10^-6^ | 4.82 |
| **PHQ9 - WSAS-5-c** | 0.85 | 0.63 - 1.08 | 6x10^-14^ | 7.51 |
| **GAD7 - WSAS-5-c** | 0.79 | 0.45 - 1.13 | 5x10^-6^ | 4.58 |
| **PHQ9 - WSAS-4-nw** | 0.83 | 0.61 - 1.04 | 5x10^-14^ | 7.54 |
| **GAD7 - WSAS-4-nw** | 0.72 | 0.38 - 1.05 | 3x10^-5^ | 4.19 |
| **WSAS-5-c - WSAS-4-nw** | 1.02 | 0.97 - 1.06 | 0 | 44.91 |

PHQ9 = depression symptoms, GAD7 = anxiety symptoms, WSAS = imputed 5-item functional impairment score used in main analysis, WSAS-5-c = complete case 5-item functional impairment score, WSAS-4-nw = 4-item functional impairment score omitting the work item. 95% CI = 95% confidence interval calculated as *r_g_* +/- (1.96*SE).

LDSC heritability estimates indicated no notable lack of power in external traits for genetic correlations (*z*-scores < 4, which are often too noisy [^11^](https://paperpile.com/c/GZBq6a/BJBWN)) but the main phenotypes, although showing significant heritability, were underpowered by this threshold (Table *II*). LDSC jack-knife analyses revealed that the genetic correlation between the PHQ9 and imputed WSAS was not significantly different from 1 (*p* = 0.15), and nor was the correlation between the GAD7 and imputed WSAS (*p* = 0.18). This is consistent with the GCTA bivariate-GREML results.

## Figure *II*. Genetic correlations (LDSC SNP-based) between the traits analysed in the present study and ten external phenotypes


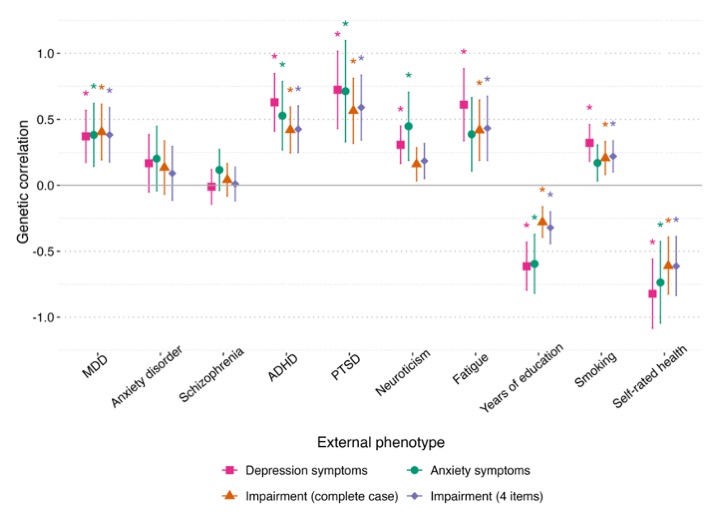


Impairment complete case = 5-item functional impairment score in participants with complete data, Impairment (4 items) = functional impairment score omitting the work item. MDD = major depressive disorder [^12^](https://paperpile.com/c/GZBq6a/BecSI), anxiety [^13^](https://paperpile.com/c/GZBq6a/PmVpz), schizophrenia [^14^](https://paperpile.com/c/GZBq6a/j8Sg0), ADHD = attention-deficit and hyperactivity disorder [^15^](https://paperpile.com/c/GZBq6a/XA5n), PTSD = post-traumatic stress disorder [^16^](https://paperpile.com/c/GZBq6a/tZWb), neuroticism [^17^](https://paperpile.com/c/GZBq6a/9k0u), fatigue [^18^](https://paperpile.com/c/GZBq6a/ZbWm), years of education [^19^](https://paperpile.com/c/GZBq6a/8uxOl), smoking [^20^](https://paperpile.com/c/GZBq6a/pqfL), self-rated health [^21^](https://paperpile.com/c/GZBq6a/MXN8P). See Supplementary Table 1 for further details of these phenotypes. Error bars represent 95% confidence intervals. * Significant at *p* < 0.005.

# Supplementary Table 4. LDSC estimated genetic correlations (*r_g_*) with external phenotypes

| **Phenotype** | **External Phenotype** | ***r_g_*** | **95% CI** | ***z*** | ***p*** |
| --- | --- | --- | --- | --- | --- |
| Depression symptoms (PHQ9) | MDD | 0.371 | 0.174 - 0.568 | 3.686 | 2x10^-4^* |
|  | Anxiety | 0.167 | -0.050 - 0.385 | 1.506 | 0.132 |
|  | Schizophrenia | -0.011 | -0.141 - 0.119 | -0.166 | 0.685 |
|  | ADHD | 0.629 | 0.411 - 0.848 | 5.643 | 2x10^-8^* |
|  | PTSD | 0.724 | 0.431 - 1.016 | 4.849 | 1x10^-6^* |
|  | Neuroticism | 0.307 | 0.165 - 0.449 | 4.242 | 2x10^-5^* |
|  | Fatigue | 0.611 | 0.338 - 0.884 | 4.384 | 1x10^-5^* |
|  | Years of education | -0.613 | -0.794 - -0.432 | -6.624 | 4x10^-11^* |
|  | Smoking | 0.322 | 0.183 - 0.461 | 4.548 | 2x10^-5^* |
|  | Self-rated health | -0.822 | -1.083 - -0.560 | -6.154 | 8x10^-10^* |
| Anxiety symptoms (GAD7) | MDD | 0.382 | 0.143 - 0.621 | 3.133 | 0.002* |
|  | Anxiety disorder | 0.202 | -0.042 - 0.447 | 1.621 | 0.105 |
|  | Schizophrenia | 0.117 | -0.039 - 0.272 | 1.471 | 0.141 |
|  | ADHD | 0.528 | 0.269 - 0.786 | 4.004 | 6x10^-5^* |
|  | PTSD | 0.713 | 0.331 - 1.096 | 3.654 | 3x10^-4^* |
|  | Neuroticism | 0.447 | 0.189 - 0.706 | 3.394 | 7x10^-4^* |
|  | Fatigue | 0.387 | 0.108 - 0.665 | 2.723 | 0.006 |
|  | Years of education | -0.595 | -0.819 - -0.371 | -5.212 | 2x10^-7^* |
|  | Smoking | 0.170 | 0.033 - 0.306 | 2.434 | 0.015 |
|  | Self-rated health | -0.736 | -1.044 - -0.427 | -4.671 | 3x10^-6^* |
| Functional impairment (WSAS) | MDD | 0.369 | 0.168 - 0.570 | 3.595 | 3x10^-4^* |
|  | Anxiety disorder | 0.122 | -0.075 - 0.319 | 1.217 | 0.224 |
|  | Schizophrenia | 0.037 | -0.083 - 0.158 | 0.607 | 0.544 |
|  | ADHD | 0.453 | 0.276 - 0.630 | 5.017 | 5x10^-7^* |
|  | PTSD | 0.568 | 0.334 - 0.802 | 4.753 | 2x10^-6^* |
|  | Neuroticism | 0.183 | 0.049 - 0.316 | 2.675 | 0.007 |
|  | Fatigue | 0.418 | 0.185 - 0.652 | 3.506 | 5x10^-4^* |
|  | Years of education | -0.329 | -0.448 - -0.209 | -5.386 | 7x10^-8^* |
|  | Smoking | 0.233 | 0.117 - 0.350 | 3.926 | 9x10^-5^* |
|  | Self-rated health | -0.605 | -0.823 - -0.387 | -5.437 | 5x10^-8^* |

MDD = major depressive disorder, ADHD = attention-deficit and hyperactivity disorder, PTSD = post-traumatic stress disorder. 95% CI = 95% confidence interval. * Significant at *p* < 0.005

# References

1. [Kroenke K, Spitzer RL, Williams JB. The PHQ-9: validity of a brief depression severity measure. *J Gen Intern Med* 2001; 16: 606–613.](http://paperpile.com/b/GZBq6a/et4B)

2. Spitzer RL, Kroenke K, Williams JB, Löwe B. A brief measure for assessing generalized anxiety disorder: the GAD-7. Archives of internal medicine. 2006 May 22;166(10):1092-7.

3. [Marks IM. *Behavioural psychotherapy: Maudsley pocket book of clinical management*. Bristol: Wright/Institute of Psychiatry Publishing, 1986.](http://paperpile.com/b/GZBq6a/mWrP)

4. Chang CC, Chow CC, Tellier LC, Vattikuti S, Purcell SM, Lee JJ. Second-generation PLINK: rising to the challenge of larger and richer datasets. Gigascience. 2015 Dec;4(1):s13742-015.

5. Taliun D, Harris DN, Kessler MD, et al. Sequencing of 53,831 diverse genomes from the NHLBI TOPMed Program. Nature. 2021 Feb 11;590(7845):290-9.

6. Das S, Forer L, Schönherr S, et al. Next-generation genotype imputation service and methods. Nature genetics. 2016 Oct;48(10):1284-7.

7. Fuchsberger C, Abecasis GR, Hinds DA. minimac2: faster genotype imputation. Bioinformatics. 2014 Oct 22;31(5):782-4.

8. Zheng J, Erzurumluoglu AM, Elsworth BL, et al. LD Hub: a centralized database and web interface to perform LD score regression that maximizes the potential of summary level GWAS data for SNP heritability and genetic correlation analysis. Bioinformatics. 2017 Jan 15;33(2):272-9.

9. [van Rheenen W, Peyrot WJ, Schork AJ, et al. Genetic correlations of polygenic disease traits: from theory to practice. Nature](http://paperpile.com/b/GZBq6a/3A9FN) Review Genetics. [2019; 20: 567–581.](http://paperpile.com/b/GZBq6a/3A9FN)

10. Yang J, Zeng J, Goddard ME, Wray NR, Visscher PM. Concepts, estimation and interpretation of SNP-based heritability. Nature genetics. 2017 Sep 1;49(9):1304-10.

11. [Bulik-Sullivan B, Finucane HK, Anttila V, et al. An atlas of genetic correlations across human diseases and traits. Nature](http://paperpile.com/b/GZBq6a/BJBWN) Genetics. [2015; 47(11):1236–1241.](http://paperpile.com/b/GZBq6a/BJBWN)

12. Wray NR, Ripke S, Mattheisen M, et al. Genome-wide association analyses identify 44 risk variants and refine the genetic architecture of major depression. Nature genetics. 2018 May;50(5):668-81.

13. Purves KL, Coleman JR, Meier SM, et al. A major role for common genetic variation in anxiety disorders. Molecular psychiatry. 2020 Dec;25(12):3292-303.

14. Trubetskoy V, Pardiñas AF, Qi T, et al. Mapping genomic loci implicates genes and synaptic biology in schizophrenia. Nature. 2022 Apr 21;604(7906):502-8.

15. Demontis D, Walters GB, Athanasiadis G, et al. Genome-wide analyses of ADHD identify 27 risk loci, refine the genetic architecture and implicate several cognitive domains. Nature genetics. 2023 Feb;55(2):198-208.

16. Stein MB, Levey DF, Cheng Z, et al. Genome-wide association analyses of post-traumatic stress disorder and its symptom subdomains in the Million Veteran Program. Nature genetics. 2021 Feb;53(2):174-84.

17. Gupta P, Galimberti M, Liu Y, et al. A genome-wide investigation into the underlying genetic architecture of personality traits and overlap with psychopathology. Nature Human Behaviour. 2024 Aug 12:1-5.

18. Deary V, Hagenaars SP, Harris SE, et al. Genetic contributions to self-reported tiredness. Molecular psychiatry. 2018 Mar;23(3):609-20.

19. Lee JJ, Wedow R, Okbay A, et al. Gene discovery and polygenic prediction from a genome-wide association study of educational attainment in 1.1 million individuals. Nature genetics. 2018 Aug;50(8):1112-21.

20. Liu M, Jiang Y, Wedow R, et al. Association studies of up to 1.2 million individuals yield new insights into the genetic etiology of tobacco and alcohol use. Nature genetics. 2019 Feb;51(2):237-44.

21. Harris SE, Hagenaars SP, Davies G, et al. Molecular genetic contributions to self-rated health. International journal of epidemiology. 2017 Jun 1;46(3):994-1009.
